# An epigenetic map of malaria parasite development from host to vector

**DOI:** 10.1038/s41598-020-63121-5

**Published:** 2020-04-14

**Authors:** Kathrin Witmer, Sabine A. Fraschka, Dina Vlachou, Richárd Bártfai, George K. Christophides

**Affiliations:** 1 Department of Life Sciences, Imperial College London, SW7 2AZ London, UK; 2 Department of Molecular Biology, Radboud University, 6525 GA Nijmegen, The Netherlands; 3 Present Address: Institute of Medical Genetics and Applied Genomics, University of Tübingen, 72076 Tübingen, Germany

**Keywords:** Development, Histone post-translational modifications, Epigenetics, Transcriptomics, Parasite development, Parasite genomics

## Abstract

The malaria parasite replicates asexually in the red blood cells of its vertebrate host employing epigenetic mechanisms to regulate gene expression in response to changes in its environment. We used chromatin immunoprecipitation followed by sequencing in conjunction with RNA sequencing to create an epigenomic and transcriptomic map of the developmental transition from asexual blood stages to male and female gametocytes and to ookinetes in the rodent malaria parasite *Plasmodium berghei*. Across the developmental stages examined, heterochromatin protein 1 associates with variantly expressed gene families localised at subtelomeric regions and variant gene expression based on heterochromatic silencing is observed only in some genes. Conversely, the euchromatin mark histone 3 lysine 9 acetylation (H3K9ac) is abundant in non-heterochromatic regions across all developmental stages. H3K9ac presents a distinct pattern of enrichment around the start codon of ribosomal protein genes in all stages but male gametocytes. Additionally, H3K9ac occupancy positively correlates with transcript abundance in all stages but female gametocytes suggesting that transcription in this stage is independent of H3K9ac levels. This finding together with known mRNA repression in female gametocytes suggests a multilayered mechanism operating in female gametocytes in preparation for fertilization and zygote development, coinciding with parasite transition from host to vector.

## Introduction

Malaria is caused by apicomplexan parasites of the genus *Plasmodium* and is transmitted to humans through bites of anopheline mosquitoes. Clinical cases and deaths decreased significantly over the past decade but began to plateau since 2015 indicating that current measures have now reached their maximum capacity and that new measures are urgently needed^1^. Transmission through the mosquito vector is a natural bottleneck in parasite development and a favorable stage for interventions aiming at malaria control and elimination. Therefore, research towards understanding parasite development in the mosquito has been intensified in recent years.

Haploid parasites infect and asexually replicate in the red blood cells (RBCs) of the mammalian host causing disease. In each replication cycle, a fraction of parasites differentiates into sexual forms called gametocytes, the stage infective to mosquitoes. Upon a bite from a mosquito, gametocytes sense the change in environment (from mammalian host to mosquito vector) and are activated to form gametes: Female and male gametocytes both exit the RBCs, and female gametocytes develop into the macrogamete by releasing messenger RNAs (mRNAs) that were stored in a messenger ribonucleoprotein (mRNP) complex for translation^2,3^. The male gametocyte, on the other hand, undergoes three rapid rounds of endomitosis and forms eight flagellated microgametes, a process called exflagellation^4^. After fertilization of the macrogamete by the microgamete, the zygote embarks on a meiotic endoreplication cycle before traversing the mosquito midgut epithelium in the form of an ookinete that upon arrival at the midgut basal side transforms into an oocyst^5^. Over two weeks, endomitotic replication in the oocyst produces hundreds of sporozoites that, upon oocyst rupture, travel to the mosquito salivary glands, ready for inoculation into the vertebrate host with the next mosquito bite.

Epigenetic regulation is crucial for parasite survival within the human host^6^. Genes involved in host-parasite interactions or coding for virulence factors or ligands involved in RBC invasion are epigenetically regulated^7,8^, while some genes involved in drug resistance are epigenetically switched on or off in an environment-dependent manner^9^.

Transcriptionally silent heterochromatin in *P. falciparum* is defined as the presence of tri-methylated histone 3 lysine 9 (H3K9me3) which is bound by HP1 (*Pf*HP1)^7,10^. Heterochromatin is largely confined to telomeric and subtelomeric regions as well as chromosome-central islands and is almost invariably associated with variantly expressed multigene protein families in *P. falciparum, P. vivax, P. chabaudi, P. berghei, P. yoelii and P. knowlesi* asexual blood stage parasites^7,10–14^, *P. falciparum* oocysts^15^ and *P. falciparum* and *P. vivax* sporozoites^15–17^. In *P. falciparum* gametocytes, heterochromatin domains expand into previously euchromatic regions harbouring genes encoding RBC remodeling proteins^14,18^, silencing genes that are used for asexual blood stage development. Euchromatic marks, on the other hand, dominate the *P. falciparum* genome: Acetylated histone 3 lysine 9 (H3K9ac) is the most investigated euchromatic mark to date and marks intergenic regions^11^. Its presence at promoter regions is a reliable predictor of gene expression in *P. falciparum* asexual blood stages^13^ and oocysts^15^, and *P. falciparum* and *P. vivax* sporozoites^15–17^. H3K4me3 is another euchromatic mark in *P. falciparum*, and while associated with active genes, levels of occupancy do not follow changes in gene expression^11–13,15^.

The binding of transcription factors to promoter regions is heavily dependent on the state of the surrounding chromatin. Members of the apicomplexan-specific ApiAP2 family of transcription factors are found to control major cell fate decision events in the parasite lifecycle, in addition to housekeeping processes^19–24^. AP2-G is the master regulator of gametocytogenesis^23,24^, activating a number of gametocyte-specific genes^25^. Similarly, the ookinete-specific AP2-O, which is itself regulated by the mRNP complex, activates transcription of over 400 genes needed for ookinete development and mosquito midgut traversal^20,26^. Three additional ookinete-specific ApiAP2 transcription factors have been identified, which play a role just before or after ookinete formation^19^.

Here, we investigate how epigenetic traits change in malaria parasites during their transition from the murine host to the mosquito vector, using *P. berghei* asexual blood stages (ABS), female (FG) and male (MG) gametocytes, and ookinetes (OOK). We confirm that heterochromatin distribution is confined to subtelomeric regions in ABS in *P. berghei*^14^. We map for the first time the heterochromatin distribution in FG, MG and OOK in any *Plasmodium* spp. and find that heterochromatin distribution remains unaltered through parasite development and between *P. berghei* lines. We find heterochromatin occupancy at only two chromosome-central genes, namely the oocyst capsule protein Cap380 and a conserved protein of unknown function (*PBANKA_0934600*). Implementing transcriptomics, we establish that variant transcription of multigene family genes occurs in *P. berghei* ABS, similar to *P. falciparum*^27^, albeit at lower levels. Additionally, we find evidence for variant multigene family transcription in MG, FG and OOK. In stark contrast to heterochromatin, the genomic distribution of H3K9ac is dynamic through parasite development: We find that while H3K9ac associates with 5′ untranslated regions (5′ UTRs) of genes, it specifically peaks around the start codon of ribosomal protein genes, a phenomenon previously not reported in any *Plasmodium* species. Consistent with previous findings in *P. falciparum* asexual blood stages^13^, H3K9ac enrichment in 5′UTRs correlates with transcript abundance in ABS in *P. berghei*. Additionally, we show for the first time that H3K9ac is a good indicator for transcript abundance in two more malaria parasite life cycle stages, namely MG and OOK. Surprisingly, we do not find a positive correlation between H3K9ac occupancy and transcript abundance in FG, suggesting a different epigenetic state of FG compared to other developmental stages. Finally, we identify four novel DNA motifs in the 5′UTR of OOK-specific genes, suggesting that transcription factors in addition to the known AP2-Os are involved in orchestrating transcription in OOK. Our study adds substantially to our understanding of epigenetic regulation of gene expression during parasite transition from the mammalian host to the mosquito vector.

## Results

### Generation of epigenetic and transcriptional profiles

To investigate chromatin changes during *P. berghei* development, we performed chromatin immunoprecipitation (ChIP) using antibodies against *P. berghei* HP1 (*Pb*HP1) as a marker for heterochromain^14^ and against H3K9ac as a marker for euchromatin. Since both *P. falciparum* and *P. berghei* histones 3 (H3) show 100% sequence conservation, we decided to use the H3K9ac antibody that has previously been used in *P. falciparum* ChIP^7,13^ in our study.

To keep samples as pure as possible and minimize contamination with another developmental stage we used three different parasite lines, one for each developmental stage, for the following reasons: Asexual blood stages (ABS) were sampled from the non-gametocyte producer PbANKA 2.33 parasite clone^28^, to rule out potential contamination with gametocytes. Male gametocytes (MG) and female gametocytes (FG) were isolated from the well-defined *820cl1m1cl1* (820) line^29^. The 820 line produces green fluorescent male gametocytes and red fluorescent female gametocytes, which can be easily sorted by flow cytometry^25,30^. As PbANKA 2.33 cannot produce ookinetes, and the 820 line is rarely used to produce ookinetes, we decided to use the well-established *507m6cl1* line^31^ for our ookinete sample (OOK). OOK were prepared from *in vitro* cultures 24 hours post gametocyte activation and subsequently purified using an antibody against the ookinete surface protein P28^32^. Chromatin was crosslinked and sheared before performing chromatin immunoprecipitation (ChIP) using either an anti-*Pb*HP1 or anti-H3K9ac antibody. The immuno-precipitated DNA and unprecipitated input as a control for each stage were purified, amplified and subjected to next-generation sequencing (NGS). To compare the ChIPseq dataset to transcript abundance, we harvested RNA in biological triplicates from the respective stages from the lines used for ABS, MG, FG and OOK ChIP to perform RNAseq. To control for any amplification bias due to the AT-rich *P. berghei* genome we included sheared genomic DNA for the RNAseq samples. A GC-plot confirms equal amplification of all regions (Fig. S1A). A summary of mapped reads for both ChIPseq and RNAseq samples is shown in Table S1.

**Figure 1:**
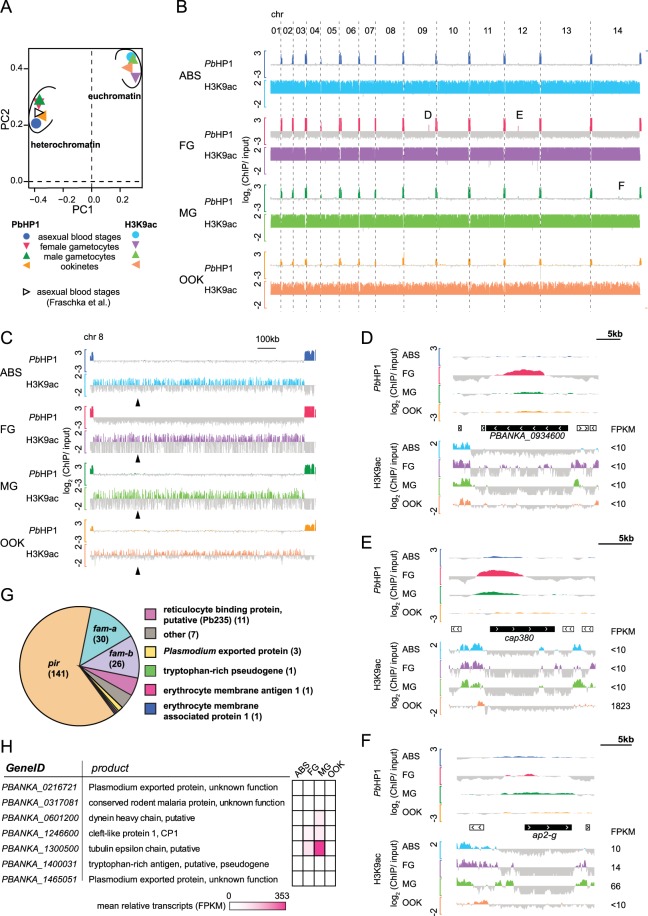
Heterochromatin distribution remains stable during malaria parasite development whereas euchromatin distribution is dynamic. (**A**) Principal component analysis of log_2_ transformed ChIP over input data. Heterochromatin and euchromatin cluster away from each other. Data from ABS from a previous heterochromatin study is included^14^. (**B**) Screenshot of heterochromatin and euchromatin distribution across all 14 *P. berghei* chromosomes for each developmental stage. Peaks correspond to log_2_ transformed data of either *Pb*HP1-ChIP/input or H3K9ac-ChIP/input. Log_2_ scale for *Pb*HP1 is (−3 to 3) and (−2 to 2) for H3K9ac, respectively. Letters highlight chromosome-central genes showing enrichment in *Pb*PH1 binding. Their close-up view is shown in (**D–F**). (**C**) Bar chart close-up view of heterochromatin and euchromatin occupancy in chromosome 8. The approximate location of the centromere (syntenically inferred from *P. falciparum*^34^) is indicated with arrowheads. (**D**,**E**) Chromosome-central genes associated with heterochromatin in this study. (**F**) *ap2-G* does not qualify as heterochromatic in our study. Black boxes show gene of interest, white boxes indicate neighbouring genes and chevrons indicate the transcriptional orientation for each gene. Peaks correspond to log_2_-transformed data of *Pb*HP1-ChIP/input (−3 to 3) and H3K9ac-ChIP/input (−2 to 2). (**G**) Pie chart of identified heterochromatic genes (including pseudogenes) grouped into gene families as described previously^36^. The number of genes in each family is shown in parentheses. (**H**) Description of seven heterochromatic genes not belonging to known multigene families. Colour bars indicate mean transcripts given as FPKM (kilobase of transcript per million mapped reads) of three independent biological replicates. ABS, asexual blood stages; FG, female gametocytes; MG, male gametocytes; OOK, ookinetes.

To get a first overview of our ChIP and RNAseq data, we perfomed principal component analysis (PCA) for each data set. Immunoprecipiated DNA of all the developmental stages clusters according to the antibody used in ChIP, suggesting that euchromatin and heterochromatin occupancies differ from each other but are similar between the different developmental stages and parasite lines (Fig. 1A). PCA of the RNAseq samples showed that transcription profiles of ABS and MG are somewhat related to each other and transcription profiles of FG are more related to OOK (Fig. S1B), as has been seen before^33^.

### Mutually exclusive profiles of heterochromatin and euchromatin

To identify genomic regions that are associated with hetero- and euchromatin during *P. berghei* development, we visualized ChIPed chromatin over input using bar plots. This approach shows that heterochromatin is confined to telomeric and subtelomeric regions of all chromosomes, except for the right arm of chromosome 4, the only subtelomeric region devoid of multigene families (Fig. 1B). The euchromatic mark H3K9ac, on the other hand, is detected in all other chromosomal regions (Fig. 1B). In contrast to most other *Plasmodium* species^14^, *P. berghei* chromosomes are largely devoid of chromosome internal heterochromatin islands and remain so during development (Fig. 1B). As shown in the example of chromosome 8 (Fig. 1C), but observed for all chromosomes and all stages, centromeres do not show enrichment in heterochromatic marks^34^.

To identify heterochromatic genes we calculated the mean *Pb*HP1 occupancy (log_2_ ratio of *Pb*HP1 ChIP to input) of the gene open reading frame (ORF) and performed hierarchical clustering of the ORF for all genes and all stages together. Using this approach, we found that only two non-telomeric and non-subtelomeric genomic genes showed clear heterochromatic profiles. The first gene identified using this approach is *PBANKA_0934600*, a gene encoding a large, conserved protein of unknown function with orthologues in all *Plasmodium* species (Fig. 1D). Transcript levels of *PBANKA_0934600* are very low (Fragments Per Kilobase of transcript per Million mapped reads (FPKM) < 10) suggesting that the gene is not expressed in any of the stages investigated here (Table S2), or indeed only expressed in a subset of cells. The latter seems to be the case, since single cell sequencing data shows that *PBANKA_0934600* is clonally variantly expressed throughout the life cycle^33^, explaining its heterochromatin status seen here. However, *PBANKA_0934600* is redundant for *P. berghei* transmission^35^ and therefore the role of this gene and why it is variantly expressed remains unclear.

The second chromosome-central heterochromatic region corresponds to *cap380* and appears to be epigenetically silenced in ABS^14^, FG and MG but not in OOK (Fig. 1E). Indeed, OOK display increased levels of relative *cap380* transcripts, which coincides with a more condensed H3K9ac occupancy within the *cap380* 5′UTR, however, it is important to note that H3K9ac occupancy in the 5′UTR of cap380 is present in all stages investigated here. *Cap380* transcription is controlled by AP2-O^26,36^ and the protein is expressed in early-stage oocysts and localizes to the oocyst capsule^37,38^. Our data provide evidence that heterochromatic silencing is an additional regulatory level of *cap380* expression, presumably preventing its premature transcription; as H3K9ac occupancy alone is no clear indication of transcriptional abundance for this gene.

A third region that showed heterochromatic marks by visual inspection but did not classify as heterochromatic using our analysis algorithm encompasses the gene encoding AP2-G (*PBANKA_1437500*; Fig. 1F). In asexual blood stages in *P. falciparum* AP2-G is epigenetically silenced by *Pf*HP1, and *Pf*HP1 eviction leads to activation of this gene^39^. Here, weak levels of *Pb*HP1 marking AP2-G in ABS can be explained by our choice of the PbANKA 2.33 parasite line. This 2.33 line carries a mutation in the *ap2-g* gene resulting in expression of a truncated, non-functional protein unable to induce gametocytogenesis^24^, thereby making its epigenetic silencing redundant.

### Epigenetic silencing of subtelomeric multigene families

Next, we asked which genes are epigenetically silenced by *Pb*HP1. Our analysis revealed that as many as 223 genes (including 61 pseudogenes) located in subtelomeric regions are significantly enriched in *Pb*HP1 binding (Fig. 1G, Table S2). Of these, 214 belong to the rodent malaria parasite (RMP) multigene family^36^. It has been previously established that *Plasmodium* heterochromatin is largely associated with gene families involved in antigenic variation and host-parasite interactions^7,14–16,18^ and our results show that the same is true for *P. berghei* gametocytes and ookinetes.

Only seven subtelomeric heterochromatic genes do not belong to RMP multigene families (Fig. 1H). They include: *PBANKA_1246600* that encodes CP1, an atypical PEXEL protein exported to discrete structures in the cytosol of infected RBCs in asexual blood stages^40^; *PBANKA_0216721* and *PBANKA_1465051* both encoding exported proteins of unknown function; *PBANKA_0317081* encoding a conserved rodent malaria parasite protein; *PBANKA_1300500* encoding a putative tubulin epsilon chain; *PBANKA_1400031*, a putative pseudogene encoding a tryptophan-rich antigen; and *PBANKA_0601200* encoding a dynein heavy chain.

*PBANKA_0601200* is one of 22 dynein-related proteins annotated in the *P. berghei* genome (Fig. S2). Dyneins are one of three cytoskeletal motor protein families in eukaryotes and made up of a protein complex of heavy, light and intermediate chain. Out of six dynein genes located close to telomeres only *PBANKA_0601200* is heterochromatic in all developmental stages in this study. However, it is not orthologous to either of two heterochromatic dynein heavy chain-encoding genes found in *P. falciparum* asexual blood stages^7^. *PBANKA_0601200* is only transcribed in MG^33^ (Fig. S2), nonetheless, its 5′UTR is occupied by H3K9ac in both male and female gametocytes, suggesting an additional layer of control. Its knockout is associated with slow ABS growth^41^.

It has previously been shown that heterochromatin boundaries expand towards the centromere in *P. falciparum* late stage gametocytes^14,18^, silencing genes involved in invasion and RBC remodelling. In contrast, we did not find any expansion of heterochromatin boundaries in either gametocyte stage or ookinetes. This highlights differences between the two *Plasmodium* species, and can be explained by the absence of the genes found on chromosome 2 in *P. berghei*. In summary, our data show that similar to other *Plasmodium* species^14,18^, subtelomeric multigene families are epigenetically silenced via *Pb*HP1 throughout *P. berghei* sexual development.

### Variant developmental expression of heterochromatic genes

**Figure 2:**
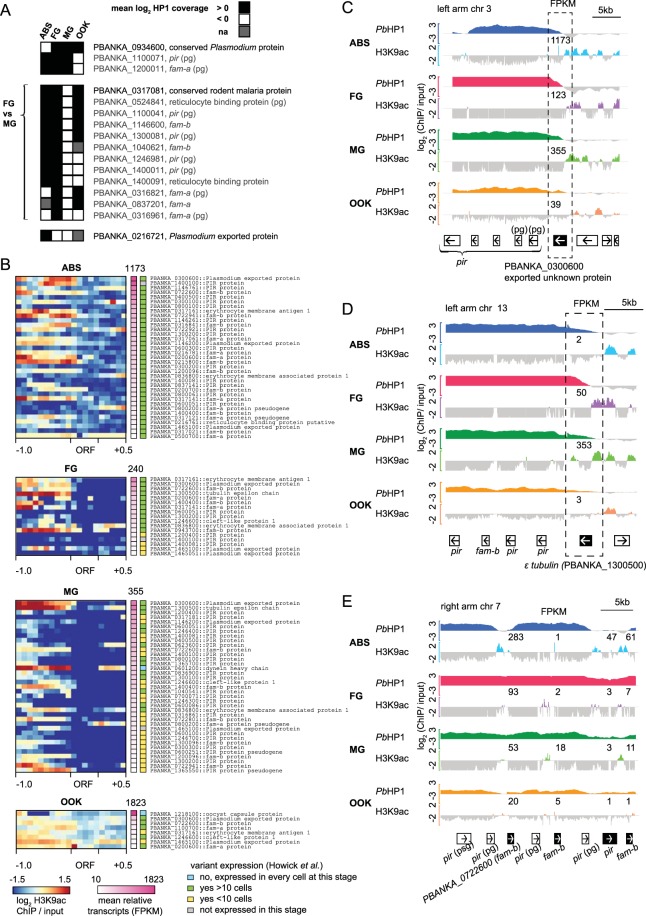
A subset of subtelomerically located genes shows signs of variant expression. (**A**) Genes with varying *Pb*HP1-occupancy through parasite development. Genes not belonging to a multigene family are highlighted with a darker colour. (**B**) Heatmap of euchromatic traits (H3K9ac-ChIP/input) of heterochromatic genes with at least 10 FPKM (fragments per kilobase of transcript per million mapped reads) for the developmental stage shown. H3K9ac enrichment for each gene locus is shown as log_2_ transformed H3K9ac ChIP/input (1000 bp upstream of ATG the ORF, and 500 bp downstream of stop codon, respectively). The left colour bar indicates mean relative transcripts (in FPKM), and the highest transcript(s) for each life cycle stage is/are named. Higher transcript numbers do not always correlate with H3K9ac occupancy within 1000 bp upstream of the start codon. The right colour bar indicates variant expression as compared to the Malaria Cell Atlas^33^. A list of all genes is found in Table S2. (**C**) *PBANKA_0300600*, an exported protein of unknown function located at the heterochromatic boundary shows both heterochromatic and euchromatic traits and is transcribed in all four life cycle stages. (**D**) *PBANKA_1300500*, the tubulin epsilon chain is both heterochromatic and euchromatic in gametocytes, and is most transcribed in male gametocytes. (**E**) Four genes within the heterochromatic region at the right arm of chromosome 7 display euchromatic traits correlating with transcription. Relative transcripts (in FPKM) for each gene are shown in numbers above ChIP-seq tracks. Black boxes show gene of interest, white boxes indicate neighbouring genes and arrows indicate the transcriptional orientation for each gene. Peaks correspond to log_2_-transformed data of *Pb*HP1-ChIP/input (−3 to 3) and H3K9ac-ChIP/input (−2 to 2). ABS, asexual blood stages; FG, female gametocytes; MG, male gametocytes; OOK, ookinetes; pg, pseudogenes.

Stochastic changes in heterochromatin distribution result in clonal variant gene expression, which forms the basis of *P. falciparum* antigenic variation^6^. In *P. berghei*, members of multigene families show variable expression independent of life cycle stage in single-cell transcriptomic data^33^. Since different parasite lines were used in our study (except FG and MG samples that derived from the same line), we could not directly determine whether differences in heterochromatin distribution are due to either the epigenetic background of the parasite lines or the developmental stage. Therefore, our comparative heterochromatic profiling would capture the sum of both. We compared the heterochromatic profiles of genes across developmental stages, and found a surprisingly small number of 16 genes with differential heterochromatin occupancy between stages/strains (Fig. 2A). This small number stands in sharp contrast to the 252 genes shown to exhibit clonal variant expression in *P. falciparum* ABS alone^27^. Thirteen of the 16 genes belonged to multigene families, and 12 showed *Pb*HP1 enrichment in FG but not in MG. Since both gametocyte samples were derived from the same line^29^, these data indicate true developmental epigenetic differences that in turn imply that heterochromatin occupancy is reorganized during gametocyte development. However, transcript abundance of these 12 genes was very low (<10 FPKM) in all lifecycle stages examined (Table S2), suggesting that absence of *Pb*HP1 occupancy may not necessarily result in an active transcriptional state.

To identify if variegated clonal expression is present in the investigated *P. berghei* life cycle stages, we arbitrarily selected all heterochromatic genes with more than 10 FPKM (mean of three replicates) in at least one developmental stage. Using this approach, we identified an additional 35 genes in ABS, 17 genes in FG, 37 genes in MG and 8 genes in OOK, respectively, which are possibly variantly expressed, as they show heterochromatic traits at the same time as transcripts (Fig. 2B). Most of these heterochromatic genes display additional euchromatic H3K9ac marks, mainly in their 5′UTR and some of them show very high transcript abundance (Fig. 2B). We compared all of these genes to the Malaria Cell Atlas data^33^ and confirmed variant expression for almost all of them in the stages investigated (Fig. 2B).

For example, despite the *PBANKA_0300600* ORF being heterochromatic, its 5′UTR is enriched in H3K9ac and the gene is highly expressed in all stages (Fig. 2C). These findings suggest that *PBANKA_0300600* is expressed in only a subset of cells. Indeed, single cell transcriptomic data^33^ shows that *PBANKA_0300600* is clonally variantly expressed in asexual blood stage parasites, male and female gametocytes and ookinetes (Fig. S3A). Similarly, epsilon-tubulin (*PBANKA_1300500*) is heterochromatic in all stages but shows euchromatic traits and high transcript abundance in both MG and FG (Fig. 2D). Again, compared to the single cell RNA transcriptomic data from Howick *et al.*^33^, epsilon tubulin is expressed in almost all male gametocytes, but shows variant expression in female gametocytes (Fig. S3B) as well as male gametocytes. Epsilon-tubulin protein marks the older of the two human centrioles upon centrosome duplication^42^ and is an essential part of the basal bodies in *Tetrahymena*^43^, but its function is unknown in *Plasmodium* species.

Another example of clonally variegated expression includes multigene family members on the right arm of chromosome 7, displaying both heterochromatin and euchromatin occupancies and high transcript abundance (Fig. 2E). This is consistent with the finding that some heterochromatic genes are transcribed by a subset of cells, effectively displaying variegated expression as shown before for members of putative exported protein families in *P. berghei*^44,45^.

We show that genes displaying hallmarks of clonally variant expression are located at the heterochromatin-euchromatin boundaries, which may facilitate clonal variation. In conclusion, we show that bulk ChIP data is able to predict variantly expressed genes defined as having both euchromatin and heterochromatin marks, as shown by our comparison to the Malaria Cell Atlas^33^.

### Distinct H3K9ac distribution in ribosomal protein genes

**Figure 3:**
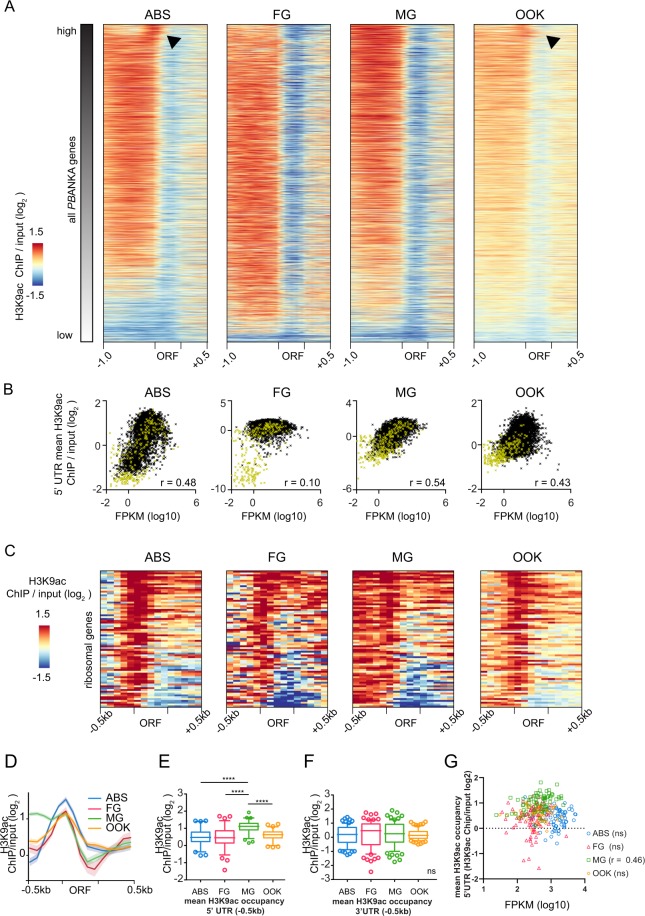
5′ UTRs of ribosomal protein genes display a distinctive H3K9ac pattern. (**A**) Heat map of H3K9ac distribution in malaria parasite development. Genes at each stage are sorted according to their relative transcription levels, with highly expressed genes on top. Arrowheads indicate a shift of H3K9ac occupancy towards the start codon. H3K9ac enrichment for each gene locus is shown as log_2_-transformed H3K9ac ChIP/input (1000 bp upstream of ATG the ORF, and 500 bp downstream of stop codon, respectively). (**B**) Scatterplot showing mean H3K9ac occupancy of 5′ UTRs (1000 bp) against transcriptional strength (mean FPKM) for each gene for each developmental stage. Spearman’s rank correlation coefficient for each scatterplot is shown (r). Genes belonging to a multigene family are highlighted in yellow. (**C**) Heat map of H3K9ac enrichment for all 74 ribosomal protein genes (40 S and 60 S). H3K9ac is enriched in a sharp peak around the start codon in ABS, FG and OOK, but less in MG. (**D**) Summary plot of the data shown in C. The mean of H3K9ac enrichment of ribosomal protein genes is shown as a bold line for each life cycle stage, standard error is shown in lighter colours. (**E**) Boxplots show mean H3K9ac-enrichment values for 5′UTRs (500 bp) of ribosomal protein genes for each stage. Whiskers indicate the 5^th^ and 95^th^ percentile, respectively. Individual symbols represent outliers. Asterisks mark significance (Wilcoxon signed rank test for matched-pairs, p ≤ 0.0001). (**F**) Boxplots show mean H3K9ac-enrichment values for 3′UTR of each ribosomal protein gene. Whiskers indicate the 5^th^ and 95^th^ percentile, respectively. Individual symbols represent outliers. Wilcoxon signed rank test for matched-pairs found no difference between the developmental stages (ns). (**G**) Scatter plot showing the correlation of the mean ribosomal protein gene transcript abundance (x-axis) against the mean H3K9ac enrichment value of the 5’UTR (500bp) (y-axis) of the same gene. The Spearman’s rank correlation coefficient is shown (r). ABS, asexual blood stage; FG, female gametocyte; MG, male gametocyte; OOK, ookinete; ORF, open reading frame.

H3K9ac is a universal histone mark associated with active promoters, including *P. falciparum* asexual blood stages, oocysts and sporozoites^11,15,16^. We investigated the relationship between H3K9ac distribution and gene transcription across the four *P. berghei* developmental stages of this study. H3K9ac enrichment in the gene ORF, 1 kb 5′UTR and 500 bp 3′UTR was examined against transcript abundance for each stage (Fig. 3A). Consistent with previous findings in *P. falciparum*, a positive correlation was detected between the gene 5′UTR H3K9ac enrichment and transcript levels in ABS^11,12^. In addition, we found that H3K9ac enrichment in the 5′UTR positively correlates with transcript abundance in MG and OOK but less so in FG (Fig. 3B), which will be investigated in more detail in the next chapter.

A shift of the peak of H3K9ac occupancy toward the gene ORF was observed for highly expressed genes, mainly in ABS and OOK (see arrowheads in Fig. 3A). Many of the genes with high expression encode ribosomal proteins. Closer investigation found that H3K9ac occupancy sharply peaks around the translation start codon of ribosomal protein genes in ABS, FG and OOK (Fig. 3C). Intriguingly, in MG, H3K9ac occupancy in these genes was extended further into the 5′UTR, resulting in no clear peak being detected around the start codon (Fig. 3D). Further analysis confirmed that the mean H3K9ac enrichment in the 500 bp upstream of the ATG (or translation start) of ribosomal protein genes was significantly different between MG and all other stages analysed (Wilcoxon signed rank test, p ≤ 0.0001), but no difference was detected between any of the other stages (Fig. 3E). As a control, the same analysis using the first 500 bp of 3′UTR sequence of these genes did not show any significant differences between any of the four stages (Wilcoxon signed rank test, p > 0.3) (Fig. 3F). This clearly shows that genes encoding ribosomal proteins exhibit different H3K9ac occupancy in comparison to all other genes in ABS, FG and OOK. Additionally, the H3K9ac intensity in the 5′UTR of ribosomal genes does not correlate with transcript abundance in ABS, FG and OOK (Fig. 3G). This also means that H3K9ac occupancy of ribosomal protein genes in MG does indeed correlate with transcript abundance.

H3K9ac occupancy is predominantly enriched around the transcriptional start site (TSS) in *P. falciparum* asexual blood stages^46^, thus we were interested to see if the shift in H3K9ac in ribosomal protein genes observed here can be explained by their TSSs lying closer to the ATG. As TSS are only mapped for *P. falciparum* but not *P. berghei*, we relied on the dataset of Adjalley and colleagues. We compared the distances from the middle of the main TSS to the ATG for ribosomal protein genes to the rest of the protein coding genes. While we found a trend for a TSS to be closer to the ATG in ribosomal protein genes compared to the rest of protein coding genes in *P. falciparum*, this was not statistically significant (unpaired t-test, P > 0.1) (Fig. S4).

Taken together, these data show that a sharp peak around the start codon is no prediction of transcript abundance in ribosomal protein genes for ABS, FG and OOK, and that this peak does not correlate with the location of the TSS. The data further show that for ribosomal protein genes, male gametocytes exhibit a different H3K9ac occupancy pattern compared to the other developmental stages investigated here and that H3K9ac intensity correlates with transcript abundance. It has been previously suggested that a shift in peak shape can indicate a different function of a gene^47^, and highly dense and narrow distributions of H3K9ac near TSSs have been associated with constitutive expression of genes involved in translation in plants^48^.

### H3K9ac enrichment does not positively correlate with transcript abundance in FG

**Figure 4:**
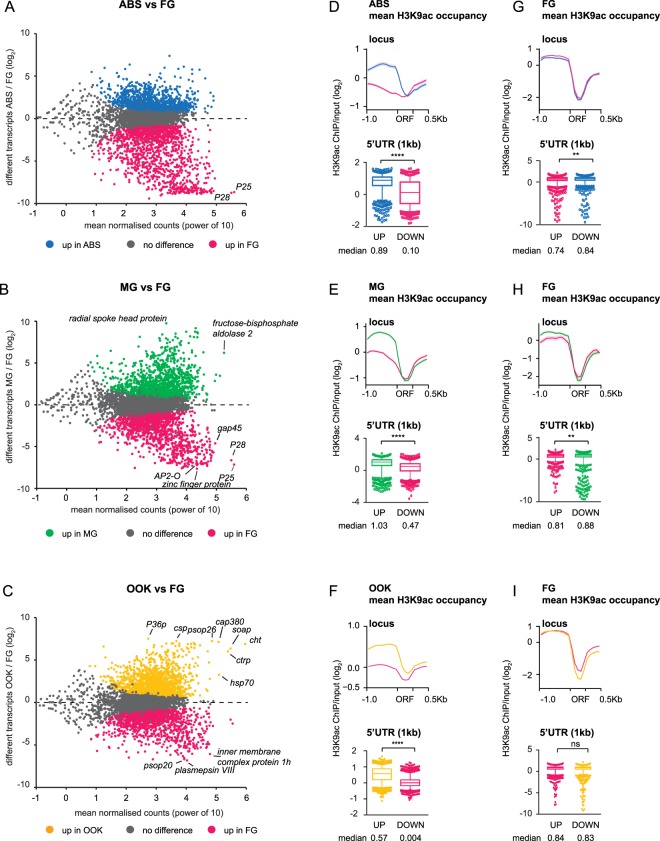
H3K9ac intensity correlates with relative transcripts in asexual blood stages, male gametocytes and ookinetes. (**A–C**) Comparative profiling of ABS, MG and OOK vs FG. Each dot represents a gene. Genes with a non-significant change in transcript abundance are shown in grey and genes that are significantly up- or downregulated are shown in their respective colour (p value adjusted for multiple testing with the Benjamini-Hochberg procedure which controls false discovery rate (FDR), here p < 0.001). (**D**) Mean H3K9ac distribution correlates with transcript levels in asexual blood stages. The upper panel shows the mean H3K9ac distribution for all genes that are either up- or downregulated in ABS vs FG. The mean H3K9ac enrichment of all loci is indicated with a solid line, and the standard error is shown in a lighter colour. 1000 bp upstream and 500 bp downstream of each gene is included. Boxplots show mean H3K9ac enrichment for the 5′ UTR for both gene groups. In ABS, (compared to FG), mean H3K9ac enrichment in the 5′UTR of a gene positively correlates with its transcript levels (Mann Whitney test, p < 0.0001). Boxes extend from the 25th to 75th percentiles, and the median is shown as a line in the middle of the box and as a number below the boxes. Whiskers indicate the 5^th^ and 95^th^ percentile, respectively. Individual symbols represent outliers. The median is shown. (**E**) In male gametocytes (compared to FG), mean H3K9ac enrichment in the 5′UTR of a gene positively correlates with its transcript levels (Mann Whitney test, p < 0.0001). Labelling is the same as in D. (**F**) In ookinetes (compared to FG), mean H3K9ac enrichment in the 5′UTR of a gene positively correlates with its transcript levels (Mann Whitney test, p < 0.0001). Labelling is the same as in D. (**G**) Mean H3K9ac distribution of in female gametocytes using the same gene groups as in A and D. Genes that are upregulated in FG (compared to ABS) are less enriched for H3K9ac in their 5′UTR of than downregulated genes in FG (Mann Whitney test, p = 0.0016). (**H**) Mean H3K9ac distribution of in female gametocytes using the same gene groups as in B and E. Genes that are upregulated in FG (compared to MG) are less enriched for H3K9ac in their 5′UTR of than downregulated genes in FG (Mann Whitney test, p = 0.0024). (**I**) Mean H3K9ac distribution of in female gametocytes using the same gene groups as in C and F. Genes that are upregulated and downregulated in FG (compared to OOK) show the same H3K9ac enrichment in FG (Mann Whitney test, non-significant (ns), p = 0.57).

We further explored the relationship between H3K9ac occupancy and transcript abundance in *P. berghei* development. For this, we identified genes that are differentially expressed between two consecutive (ABS and FG, FG and OOK) or opposite (FG and MG) developmental stages and examined their H3K9ac occupancy for each of the two stages (Fig. 4A–C). Genes upregulated in ABS (1426), MG (1523) or OOK (1641) compared to FG exhibit greater H3K9ac enrichment in their 5′UTR (1000 bp upstream of the start codon) in each respective stage than genes downregulated in any of these three stages (1429, 1518 and 1534, respectively) compared to FG (Fig. 4D–F). These results indicate that H3K9ac occupancy in the gene 5′UTR is a good predictor for relative transcript abundance in ABS, MG and OOK. Surprisingly, when examining the same gene groups in FG, upregulated genes in FG showed less (FG vs. ABS and FG vs. MG) or the same (FG vs. OOK) H3K9ac enrichment than/as downregulated genes (Fig. 4G–I).

**Figure 5:**
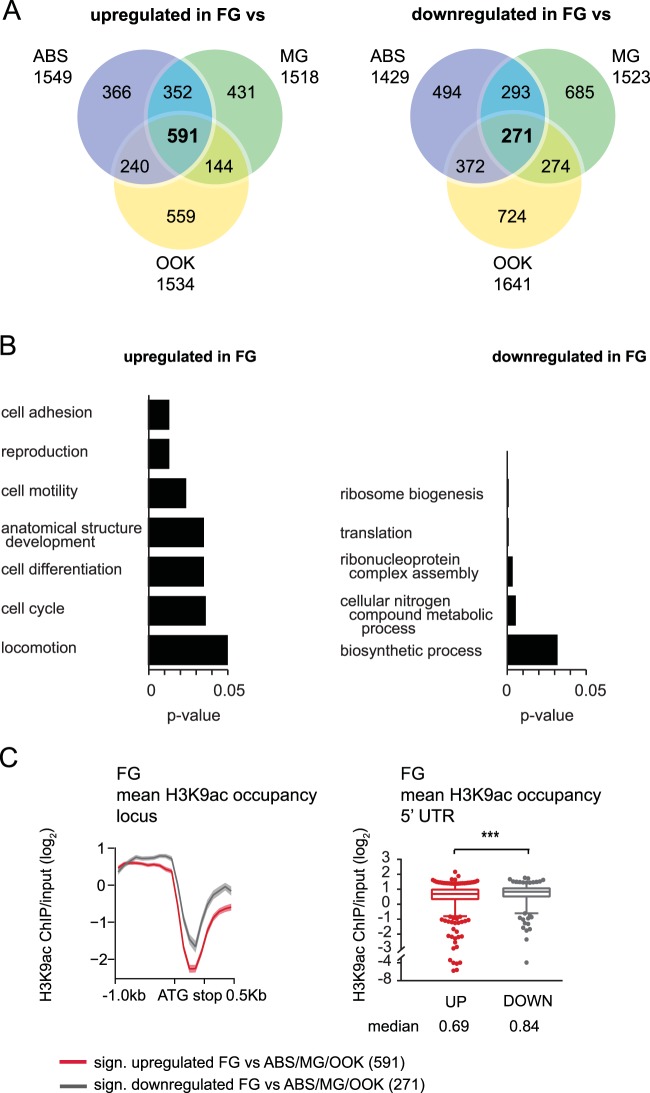
H3K9ac enrichment in the 5′UTR of a gene does not positively correlate with its relative transcripts in female gametocytes. (**A**) Venn diagram of all genes that are significantly up- or downregulated in female gametocytes compared to asexual blood stages, male gametocytes and ookinetes, respectively. (**B**) Enriched GO-terms (slim) of both up- and downregulated genes in female gametocytes compared to all other life cycle stages. Each respective p-value is indicated. The full list of genes and GO-terms can be found in Table S4. (**C**) H3K9ac enrichment in female gametocytes for female gametocyte-specific genes. The left panel shows H3K9ac enrichment in female gametocytes for genes that are significantly up- or downregulated in female gametocytes only. The mean H3K9ac enrichment of all loci is indicated with a solid line, and the standard error is shown in a lighter colour. 1000 bp upstream and 500 bp downstream of each gene is included. Boxplots show mean H3K9ac enrichment for the 5′ UTR for both gene groups. Mean H3K9ac enrichment in the 5′UTR of a gene negatively correlates with its transcript levels (Mann-Whitney test, p-value=0.0009). Boxes extend from the 25th to 75th percentiles, and the median is shown as a line in the middle of the box. Whiskers indicate 5 and 95 percentiles, respectively, and outliners are individual dots. The median is shown.

We further investigated the lack of positive correlation between H3K9ac occupancy and transcript abundance in FG by generating two high-confidence lists of genes that are either upregulated (591 genes) or downregulated (271 genes) in FG compared to all other stages, respectively (Fig. 5A and Table S3). Gene Ontology (GO) analysis using the GO slim version showed that the former list includes genes involved in cell adhesion, reproduction, motility, differentiation, cell cycle and locomotion, all of which are characteristic of cells preparing for fertilization and development into the motile ookinete stage (Fig. 5B and Table S4). In contrast, downregulated genes in FG are involved in ribosome biogenesis and translation, suggestive of a translationally less active FG stage compared to the other three stages. FG are known to produce and store mRNAs that are translationally repressed^2^. Similar to before, these downregulated FG genes show significantly higher H3K9ac enrichment in their 5′UTRs than genes that are upregulated in FG (Fig. 5C).

Together, these data indicate that H3K9ac, a histone mark previously associated with active promoters in *P. falciparum* asexual blood stages^12,13^ and oocysts^49^ as well as *P. falciparum*^15,16^ and *P. vivax*^17^ sporozoites, also associates with transcript abundance in *P. berghei* ABS, MG and OOK. Additionally, we show that H3K9ac occupancy does not directly correlate with transcript abundance in FG in *P. berghei*. Instead, it appears that H3K9ac marks the promoters of most genes in FG, irrespective of their transcript abundance.

Post-transcriptional regulation is known to occur in female gametocytes, where transcripts are stabilized by RNA binding proteins DOZI and CITH, and are poised for translation^2,3,50^. Therefore, large amounts of maternal transcripts accumulate in the female gametocyte not necessarily reflecting transcriptional states of these genes. To test if the lack of positive correlation between H3K9ac occupancy and transcript abundance in FG may be caused by poised transcripts, we compared the H3K9ac occupancy of translationally repressed genes to the sum of all genes (Fig. S5A). We find that mRNA of genes that are translationally repressed by the DOZI-complex show lower H3K9ac occupancy compared to all euchromatic genes (all genes minus multigene family genes, minus *Pb*HP1 occupied genes) (Fig. S5B**)**, confirming that high transcript abundance due to storage does not positively correlate with H3K9ac occupancy in FG.

After fertilization, ookinete-specific transcripts are transcribed *de novo*, activated by the ookinete AP2 transcription factor AP2-O^20,26^. To test if H3K9ac enrichment in FG predicts future transcriptional activity, we compared H3K9ac occupancy of AP2-O regulated genes to the sum of all genes (Fig. S5C). We found that H3K9ac enrichment in FG is unrelated to future transcriptional activity, as AP2-O-regulated genes display the same H3K9ac enrichment compared to all euchromatic genes in the *P. berghei* genome (Fig. S5D). Taken together, these findings indicate a H3K9 hyperacetylated genome in FG where H3K9ac does not predict transcript abundance. Further experiments will be needed to determine if and which histone modifications are predictive of transcript abundance in FG.

### Novel and known DNA motifs control ookinete gene expression

In most metazoans as well as in flowering plants, maternally deposited proteins and mRNAs are responsible for directing early stages of development post fertilization, while *de novo* transcription is resumed via specific transcription factors, a phase called maternal-to-zygotic transition (MZT). In *Plasmodium*, maturation of the fertilized female gamete to the ookinete is dependent upon both de-repression of maternal mRNAs^2,50^ and *de novo* transcription mediated by ookinete-specific transcription factors such as AP2-O^19,20,26^.

**Figure 6:**
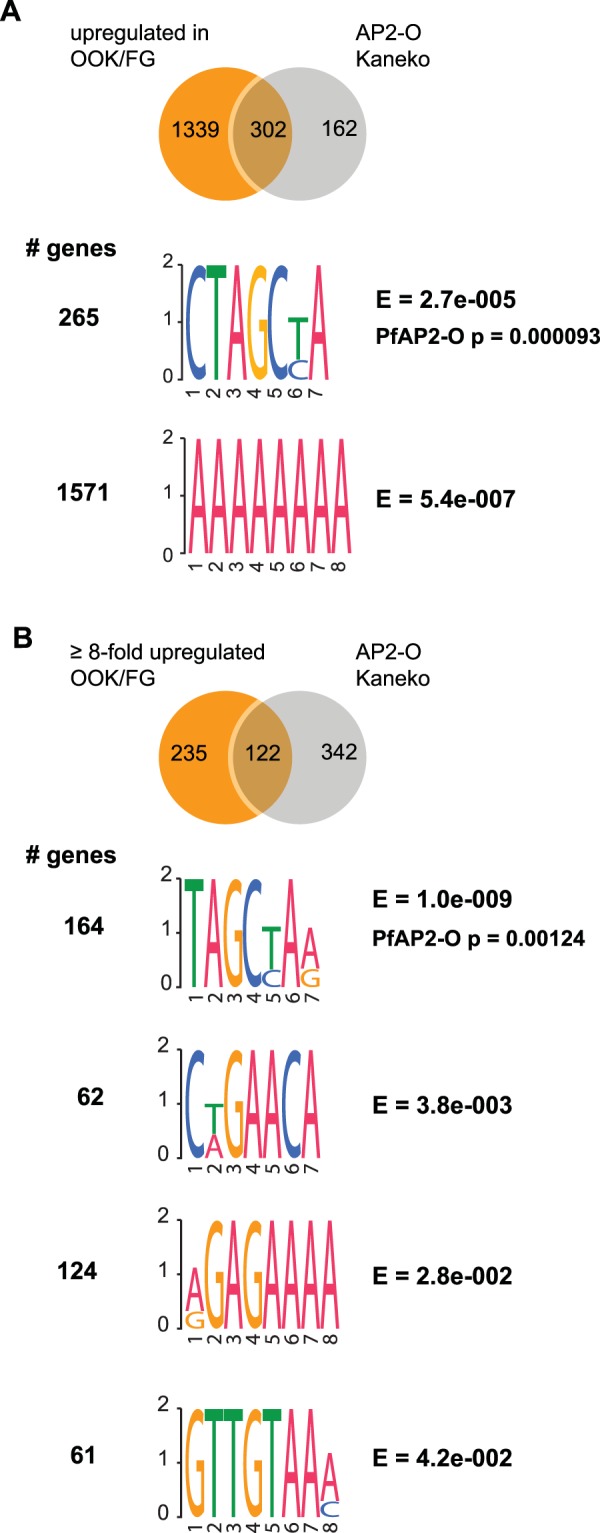
Novel and known DNA-binding motifs enriched in the 5′ UTR of ookinete-specific genes. (**A**) Venn diagram of genes that are significantly up regulated in ookinetes vs female gametocytes and their overlap with AP2-O genes from Kaneko *et al.*^26^. DNA motifs significantly enriched in in the 5′ UTR of all significantly upregulated ookinete genes compared to the 5′ UTR of all genes. The number of genes exhibiting each motif is shown as well as the E-value for each motif. The AP2-O motif is highly enriched. (**B**) Venn diagram of genes that are at least 8-fold significantly up regulated in ookinetes vs female gametocytes and their overlap with AP2-O genes from Kaneko *et al.*^26^ DNA motifs significantly enriched in the 5′ UTR of 8-fold upregulated ookinete genes compared to the 5′ UTR of all genes. The number of genes exhibiting each motif is shown as well as the E-value for each motif. The AP2-O motif is highly enriched.

We further investigated the mechanisms of MZT in *P. berghei* starting with genes that are differentially upregulated in OOK compared to FG (Fig. 4C), and compared them to 464 genes previously annotated as controlled by AP2-O^26^ (Fig. 6A). Of the 1641 ookinete-specific genes, only 302 qualified as AP2-O controlled (Fig. 6A), suggesting that not all AP2-O controlled genes have been annotated to date and/or that additional transcription factor(s) might be involved in ookinete-specific transcription. Therefore, we searched for DNA-motifs^51^ that are significantly enriched in the 5′UTRs of all 1641 OOK-enriched genes and found two highly significantly enriched motifs: the AP2-O CTAGCT/CA motif^20^ present in 265 genes, and the AAAAAAAA motif found in as many as 1571 genes (Fig. 6A).

We repeated the same search using a more stringent list of 357 genes that are at least 8-fold upregulated in OOK compared to FG; and only 122 qualified as controlled by AP2-O (Fig. 6B). Four DNA-motifs were identified, the most significant of which again resembles the PfAP2-O motif (TAGCT/CAA/G) and was found in almost half of all genes (164). The other three novel motifs were CT/AGAACA (in 62 genes), A/GGAGAAAA (in 124 genes) and GTTGTAAA/C (in 61 genes).

These data confirm previous findings that AP2-O is a master regulator of *de novo* transcription and MZT in *Plasmodium* and indicate the possibility of the existence of additional transcription factors involved in this process. It is important to note that while additional OOK transcription factors have been described^19,33^, none of the published DNA-motifs matches those found here.

## Discussion

Our study provides new insights into the epigenetic regulation of *Plasmodium* gene expression during its developmental transition from asexual to sexual and zygotic stages, which occurs as the parasite moves from its vertebrate host to the insect vector and is essential for parasite transmission.

Epigenetic silencing has been associated with key strategies of parasite development and environmental adaptations. Our data reveal that HP1-mediated epigenetic silencing remains largely unchanged during this transition and confined to chromosomal subtelomeric regions. Mapping *Plasmodium* subtelomeric genes is very challenging as most genes belong to multigene families with high levels of sequence identity between them. Indeed, as many as 51 heterochromatic genes could not be mapped to *P. berghei* chromosomes in our analysis: 14 *fam-a*, 8 *fam-b*, 1 fam-c, 27 *pir*, and one encoding a tryptophan-rich antigen.

It is important to note that the study by Fraschka *et al.*^14^ analyzed *Pb*HP1 occupancy in *P. berghei* ANKA asexual blood stages. Their strain is the parental strain of the PBANKA 2.33 line used in this study. Comparison between the two studies showed that 177 heterochromatic genes are shared between the two studies, 46 are specific to our study and 14 are specific to the study of Fraschka *et al.* (Fig. S6A). Whereas the vast majority of differences pertain to subtelomeric regions, where our analysis appears to be more sensitive, the combination of the two studies identified four non-subtelomeric heterochromatic genes, of which only *cap380* encoding an oocyst capsule protein is detected in both studies. On the one hand, *PBANKA_0934600* that encodes a protein of unknown function was only detected in our analysis but a closer visual inspection of the profiles obtained by Fraschka *et al.*^14^ shows extensive occupancy of the gene by *Pb*HP1 (Fig. S6B). On the other hand, *ap2-g* and *ap2-sp3/ap2-tel* are not detected as heterochromatic in our analysis but visual inspection indicates increased *Pb*HP1 occupancy (Fig. S6C). These data suggest that automated detection of moderate heterochromatin enrichment remains a challenge and that conclusions about absence of epigenetic silencing in such regions should be treated with caution. Nonetheless, the two studies together reveal that *P. berghei* heterochromatin formation, maintenance and inheritance are largely hardwired throughout development.

It has been previously proposed that epigenetic reprogramming or resetting of the expression of genes involved in parasite virulence occurs during parasite passage through the vector^52,53^. However, no such evidence for a reset of virulence is detected pre- and post-meiotically in our study, which could be explained by two scenarios. Firstly, such reset is likely to start immediately after nuclear fusion as in higher eukaryotes^54^ and thus may have been missed by our study design. Secondly, any such presumed reset may involve different epigenetic marks than those examined here. Finally, the virulence-reset hypothesis is based on parasites serially maintained in rodents, which are known to become more virulent. Therefore, it is probable that the observed phenotype by Spence *et al.* is based on ill-managed heterochromatin maintenance undetectable by our study.

Gametocytes are poised cells that are rapidly activated and transform into respective gametes upon ingestion by a mosquito. In *P. falciparum*, heterochromatin boundaries are shown to expand outside telomeres as the parasite lifecycle progresses from ABS to gametocytes^14^. However, our data do not show any major differences in heterochromatin distribution between the various *P. berghei* lifecycle stages examined. This result can be explained by the fact that genes that become heterochromatic in *P. falciparum* gametocytes and are involved iRBC remodeling (such as knob formation) have no clear orthologues in *P. berghei* and highlights differences of heterochromatin maintenance between the two parasite species.

Male and female gametocytes are characterized by specific transcriptomes and proteomes^25,30,55,56^. However, in our analysis, differential heterochromatin occupancy between these two stages is limited to 12 genes, suggesting that epigenetic gene silencing is not a key regulatory mechanism of differential gene expression between male and female gametocytes. In addition, all these genes are located in subtelomeric regions and 11 of them belong to a multigene family, and thus some of the detected differences may be due to the method’s sensitivity. It remains to be seen whether any of the 12 genes show true expression differences between male and female gametocytes and relate to sex-specific functions.

Clonally variant expression of subtelomeric gene families has been extensively examined in *P. falciparum*^27,57,58^. In contrast, variant gene expression in other *Plasmodium spp*. is less well investigated^33^. Due to the nature of clonal variant expression, its identification is always hampered by the fact that bulk RNA sequencing captures the sum of all the transcripts in the population, hence the identification of variant gene expression traditionally involved the analysis of clones derived from the same starting population^27^ and more recently, single cell sequencing^33^. Here, our approach of combining bulk RNA sequencing data with euchromatic and heterochromatic marks identified heterochromatic genes that are transcribed and sometimes even display a euchromatic environment, suggesting that they are variantly expressed withing the population. Indeed, single cell sequencing data confirmed that most of these genes are variantly expressed in a different *P. berghei* line^33^. Therefore, combining euchromatic, transcriptional and heterochromatic traits can be used to predict variantly expressed genes as an alternative to single cell sequencing.

In line with previous findings^33^, we confirm that the number of variantly expressed genes is significantly lower in *P. berghei* than in *P. falciparum*. We currently do not have any explanation for this finding but can provide two hypotheses. Firstly, it is possible that by keeping *P. berghei* in its “unnatural” host of outbred mice under controlled conditions decreases its active multigene repertoire, and as a result, *P. berghei* fix on a low number of genes to be expressed, similar to *P. falciparum* under *in vitro* culture conditions^58^. Secondly, it is known that in the rodent malaria parasite *P. chabaudi*, the number of expressed *cir* genes is highest directly after mosquito transmission and decreases with multiple passages between mice^53^. It is possible that our observations of the low number of variantly expressed genes are based on this phenomenon.

In several *Plasmodium* species and life stages, it is now proven that the occupancy of the euchromatic mark H3K9ac in the 5′UTR of a gene correlates with its transcription^11,13,15–17^. Here we show for the first time that the same is true for *P. berghei* asexual blood stages. Additionally, for the first time for any *Plasmodium spp*., we reveal that the correlation between H3K9ac and gene expression also applies to male gametocytes and ookinetes.

Here, the peak shape of H3K9ac occupancy was further investigated. We show that ribosomal protein genes display a very specific H3K9ac pattern peaking around the translational start codon in asexual blood stages, female gametocytes and ookinetes. This pattern is irrespective of transcript intensity but is connected to the gene group instead. Additionally, using mapped TSS from *P. falciparum*^46^, we do not find that TSSs of ribosomal protein genes to be closer to the ATG than TSSs from other genes. A similar sharp H3K9ac peak around the TSS has been found in *A. thaliana* for genes involved in translation^48^ which could explain our findings. We have currently no explanation why this pattern is not found in male gametocytes.

A striking difference between male and female gametocytes is the level of H3K9ac occupancy in the 5′UTR of genes, which correlates with transcript abundance in male but not in female gametocytes. Thus it appears that gene transcript abundance in female gametocytes is independent of H3K9ac levels. We propose that the female gametocyte is a stage of epigenetic remodeling that involves universal marking of gene promoters with H3K9ac. At the same time, genes that are involved in ribosome biogenesis and translation are downregulated, a phenomenon that together with translational mRNA repression by the DOZI-complex suggests that a multilayered mechanism regulating zygotic development operates in *Plasmodium*. It will be interesting to see how other euchromatic marks for example H3K4me3 are distributed in female gametocytes. In mice, H3K4me3 occupancy usually remains narrowly restricted to promoter regions but its occupancy expands during oocyte development^59–61^. It is possible that a similar occupancy shift occurs in female gametocytes.

Mature oocytes in mammals, flies and worms are transcriptionally silent when passing through meiosis I preceding fertilization^62^. Additionally, early embryonic development depends solely on maternally deposited proteins and RNAs and coincides with low or undetectable transcription^63^. Zygotic transcription often resumes after several hours or days post fertilization, a process called maternal-to-zygotic transition accompanied by zygotic genome activation, and in flies, for example, is controlled by a master transcription factor that is not necessarily associated with H3K9ac^64^. Additionally, epigenetic reprogramming occurs before and after fertilization, ensuring the transition from a highly specific cell type back to a totipotency state able to form a new organism. However, direct comparison of malaria parasite female gametocytes to oocytes of multicellular organisms is complicated by the fact that malaria parasites undergo meiosis directly after fertilization^65^, in contrast to metazoan cells where meiosis precedes fertilization. Nonetheless, it is tempting to hypothesise that, similar to higher eukaryotes, (i) the female gametocyte is transcriptionally poised and (ii) transcription in the ookinete is resumed only from a selected set of genes. While there is no evidence so far that the female gametocyte is transcriptionally poised, there is evidence for the second part of the hypothesis. Firstly, when expression of a fluorescent reporter protein is controlled by an ookinete-specific gene promoter the protein is detected from both the maternal and paternal genomes in the first 24 hours after fertilization^66^. Secondly, if expression of a fluorescent reporter protein is controlled by an housekeeping gene promoter the protein is detected only from the maternal genome^66^. This could either mean that the paternal genome is silenced, or more likely, that both parental genomes are transcriptionally poised, and the detected expression stems from inherited maternal mRNA.

Two histone variants associated with active promoters in *P. falciparum* ABS parasites (H2A.Z and H2B.Z) ^13,67^ are significantly downregulated in female gametocytes in this study (Table S3). As mRNA is stored in the RNP complex in female gametocytes, we do not know which mRNA is translated into protein and which mRNA is translationally poised at this stage. Thus,it will be interesting to see if either female gametocytes are have a low occurrence of H2A.Z and H2B.Z, or if these histone variants are not deposited into the chromatin during the early development of the zygote. Proteomic studies identified both H2A.Z and H2B.Z in female gametocytes in both *P. berghei* and *P. falciparum*^55,56^, pointing towards the latter possibility. Supportively, in mice, H2A.Z remains undetectable in embryonic chromatin before the late 2-cell stage^68^. Thus, we propose a scenario where the female gametocyte is transcriptionally poised, and where transcription of stage-specific genes is resumed in the ookinete stage via AP2-O, and potentially by additional transcription factors, as we and others^19,33^ identified additional DNA motifs abundant in ookinete-specific genes. Still, it is important to note that these newly identified motifs need experimental validation in future studies.

## Material and Methods

### Ethics statement

All animal experimental procedures were reviewed and approved by the Imperial College London Animal Welfare and Ethical Review Body (AWERB) and the United Kingdom Home Office. These procedures were in accordance with the Animal Scientifics Procedures Act 1986, under the UK Home Office Licenses PLL70/7185 and PPL70/8788.

### Plasmodium berghei maintenance

*P. berghei* clone *ANKA 2.33* (for asexual blood stages)^28^ and the *507m6cl1 (c507)* line (for ookinetes)^31^ were maintained in 6–8 week old female Tuck’s Ordinary (TO) (Harlan, UK). The *820cl1m1cl1 (wt-fluo)* line (for gametocytes)^29^ was maintained in 6–8 week old female CD1 mice (Harlan, UK).

### Chromatin extraction and fragmentation

Asexual blood stage parasites were harvested via heart puncture and passed through a Plasmodipur filter (Europroxima) to remove leucocytes, resuspended in RPMI-1640 medium (Sigma-Aldrich) and crosslinked with 1% formaldehyde in PBS for 10 min at 37 °C. Crosslinking was quenched adding glycine to an end concentration of 0.125 M. RBC were then lysed with 0.15% saponin (in PBS) on ice for 5–10 minutes. To obtain nuclei the resulting parasite pellet was lysed with cell lysis buffer (20 mM Hepes, 10 mM KCl,1 mM EDTA, 1 mM EGTA, 0.65% NP-40, 1 mM DTT, 1x protease inhibitor (Roche)). The pellet was resuspended in sonication buffer (1% SDS, 50 mM Tris pH8, 10 mM EDTA, 1x protease inhibitor (cOmplete, Mini, EDTA-free, Roche)) and sheared for 25 minutes (30 sec ON, 30 sec OFF; settings HIGH) using a Bioruptor Plus sonication device (Diagenode) to obtain DNA fragments of around 100–300 bp.

The method for the purification of gametocytes was modified from^69^. Briefly, mice were pretreated by intra peritoneal injection of 0.2 ml phenylhydrazine (6 mg/ml in PBS) to stimulate reticulocyte formation two days prior to infection with parasites. Gametocyte-enriched blood was harvested via heart puncture and blood was immediately resuspended in 4 °C coelenterazine loading buffer (CLB) (1x PBS, 20 mM HEPES, 20 mM glucose, 4 mM sodium bicarbonate, 1 mM EDTA, 0.1% BSA in PBS, pH 7.24–7.31) and magnet-purified using D Columns on a SuperMACS II Separator (Miltenyi Biotec). Magnet-purified parasites were crosslinked in 1% formaldehyde for 10 minutes at 37 °C, quenched with glycine to an end concentration of 0.125 M and resuspended in FACS buffer (PBS with 2 mM HEPES, 2 mM glucose, 0.4 mM NaHCO3, 0.01% BSA, 2.5 mM EDTA). Male and female gametocytes were sorted according to color (GFP for males and RFP for females) on a FACSAria III with a 70uM nozzle at 4 °C. Purified gametocyte pellets were lysed in 150ul sonication buffer and chromatin prepared as above. FACS was performed with four biological replicates, and a total number of 3.1E7 female gametocytes and 2.2E7 male gametocytes were used for ChIP, respectively.

Ookinetes were cultured *in vitro* as described^70^.Briefly, mice were pretreated by intra peritoneal injection of 0.2 ml phenylhydrazine (6 mg/ml in PBS) to stimulate reticulocyte formation two days prior to infection with parasites. Gametocyte-enriched blood was harvested via heart puncture and blood was immediately resuspended in 30 ml of ookinete medium (RPMI-HEPES complemented with 100uM xanthurenic acid, 200 uM hypoxanthine and 10% BSA, pH7.4) and incubated at 21 °C for 24 hours. After 24 hours, ookinetes were magnet-purified using 1ul monoclonal anti-P28 antibody 13.1^32^ coupled to 10ul magnetic beads (Dynabeads M-280 Sheep Anti-Mouse IgG). Purified ookinetes were crosslinked in 1% formaldehyde for 10 minutes at 37 °C, quenched with glycine to an end concentration of 0.125 M. Purified ookinetes pellets were lysed in 150ul sonication buffer and chromatin was prepared as above. Two ookinete cultures were pooled for the ChIP to obtain enough material, with female to ookinete conversion rates of 66% and 71.5%, respectively.

### Chromatin immunoprecipitation

Antibodies used for ChIP were rabbit anti H3K9ac (Diagenode Cat# C15410004, RRID:AB_2713905) and rabbit anti-*Pb*HP1^14^. 1ug antibody was incubated with up to 500 ng chromatin in ChIP buffer (5% TritonX-100, 750 mM NaCl, 5 mM EDTA, 2.5 mM EGTA, 100 mM Hepes) at 4 °C over night. The next day, 50ul Protein A Dynabeads (Fisher 10001D) were added and further incubated for 1–2 h. After washing with buffers containing 100, 150 and 250 mM NaCl, immunoprecipitated DNA was eluted and purified using PCR minelute purification columns (Qiagen).

For each antibody several ChIP reactions were performed in parallel to obtain sufficient amount of DNA for ChIP-seq: (asexual blood stages (3xH3K9ac and 8×*Pb*HP1); female/male gametocytes (3x H3K9ac and 6×*Pb*HP1); ookinetes (2x H3K9ac and 5×*Pb*HP1)).

The following number of biological replicates were pooled to obtain enough material for ChIP: ABS (1); FG (4), MG(4), OOK (2).

### ChIP sequencing

For each sequencing library up to 10 ng of ChIP or input DNA were end-repaired, extended with 30 A-overhangs and ligated to barcoded NextFlex adapters (Bio Scientific) as described previously^71^. Libraries were amplified (98 °C for 2 min; four cycles 98 °C for 20 sec, 62 °C for 3 min; 62 °C for 5 min) using KAPA HiFi HotStart ready mix (KAPA Biosystems) and NextFlex primer mix (Bio Scientific) as described^72^. 225–325 bp fragments (including the 125 bp NextFlex adapter) were size- selected using a 2% E-Gel Size Select agarose gel (Thermo Fisher Scientific) and amplified by PCR for ten (asexual blood stages and ookinetes) or eleven (female and male gametocytes) cycles under the same condition as described above. Library purification and removal of adapter dimers was performed with Agencourt AMPure XP beads in a 1:1 library:beads ratio (Beckman Coulter). ChIP-seq libraries were sequenced for 75 bp single-end reads using the NextSeq. 500/550 High Output v2 kit (Illumina) on the Illumina NextSeq. 500 system.

Sequencing reads were mapped against the *P. berghei* ANKA reference genome v3 using BWA samse (v0.7.12-r1039)^73^ (and filtered to mapping quality ≥15 (SAMtools v1.2)^74^. Only uniquely mapped reads were used for further analysis. The PCA plot was calculated using default settings in the DeepTools2 suite^75^ using non-overlapping 1000 bp bins.

### *Pb*HP1 analysis

The bam files containing mapped reads from each ChIP were normalised against their input using *bamcompare* in DeepTools2^75^. For *Pb*HP1, the default settings were used with the following changes: 100 bp bin size, 0.01 pseudocount and 1000 bp smoothing.

The mean of the log2 *Pb*HP1/input ratio was then extracted for each gene, making each gene fit 500 bp using one 500 bp bin using *computematrix* in DeepTools2^75^ with default settings with the following changes: missing values = 0 and skip 0. The resulting *bigwig* files were hierarchically clustered with average linkage and Euclidean distance as similarity metric in Cluster3.0^76^. The resulting heatmap and tree was inspected in Treeview^77^.

The multigene family list is based on Table S4 from Otto *et al.*,^36^ and we updated gene IDs to *P. berghei* genome version 3 and found 379 genes.

For visualising our data next to the data from Fraschka *et al.*^14^ we used the author’s *Pb*HP1 over input bedgraph file from the GEO database (GSE102695) and viewed it in IGV^78^. Chromosome plots were drawn using Phenogram^79^.

### H3K9ac analysis

The bam files containing aligned reads from each ChIP were normalised against their input using *bamcompare* from the *DeepTools2 suite*^75^. For H3K9ac, default settings were used with the following changes: bin size 50 bp, 0.01 pseudocount, 100 bp smoothing. Analyses using heatmaps and summary plots were performed using the deeptools2^75^ suite on usegalaxy.org as well as usegalaxy.eu.

For the ribosomal subunit genes, we used the *P. berghei* orthologues from *P. falciparum* reported in^80^. For statistical analysis and drawing of boxplots, we used Graphpad Prism 7.03. Wilcoxon matched-pairs signed rank test was performed on each data set and P-values <0.01 are reported, with (****) P ≤ 0.0001.

### Motif identification

5′ UTRs were extracted from *PB*ANKA genome version 3. Sequences between annotated genes were attributed to the nearest gene as follows: For head-to-head genes, the 5′ UTRs were split 1:1. For tail-to-head genes, the sequence was split 1:2 (1/3 was assigned as 3′ downstream region to the gene ending and 2/3 was assigned as 5′ UTR to the gene starting). To find motifs enriched in ookinete-expressed genes, we used DREME (v 5.0.0)^51^ with default settings locally on the MEME suite^81^.

We used the build-in Gene Ontology tool of PlasmoDB with default settings to identify enriched GO terms^82^.

### RNA preparation

Asexual blood stage parasites were harvested via heart puncture and resuspended in 15 ml RPMI-HEPES and passed through a Plasmodipur filter (Europroxima) to remove white blood cells. Parasite/RBC pellet was lysed with 10 ml RBC-lysis buffer (150 mM NH_4_Cl, 10 mM KHCO_3_, 1 mM EDTA) for 20 min on ice. Parasite-pellets were washed once in ice-cold PBS and lysed in 500ul TRIzol (Invitrogen) and stored at −80 °C.

The method for the purification of gametocytes was modified from^69^. Briefly, mice were pretreated by intra peritoneal injection of 0.2 ml phenylhydrazine (6 mg/ml in PBS) to stimulate reticulocyte formation two days prior to infection with parasites. Gametocytes were harvested via heart puncture and blood was immediate resuspended in 4 °C coelenterazine loading buffer (CLB) (1x PBS, 20 mM HEPES, 20 mM glucose, 4 mM sodium bicarbonate, 1 mM EDTA, 0.1% BSA in PBS, pH 7.24–7.31) and magnet-purified using D Columns on a SuperMACS II Separator (Miltenyi Biotec). Magnet-purified parasites were resuspended in FACS buffer (PBS with 2 mM HEPES, 2 mM glucose, 0.4 mM NaHCO3, 0.01% BSA, 2.5 mM EDTA). Male and female gametocytes were sorted according to color (GFP for males and RFP for females) on a FACSAria III with a 100uM nozzle at 4 °C. Purified gametocyte pellets were lysed in 500ul TRIzol (Invitrogen) and stored at −80 °C.

Ookinetes were cultured *in vitro* as described^70^. Briefly, mice were pre-treated by intra peritoneal injection of 0.2 ml phenylhydrazine (6 mg/ml in PBS) to stimulate reticulocyte formation two days prior to infection with parasites. Gametocytes-enriched blood was harvested via heart puncture and blood was immediately resuspended in 30 ml of ookinete medium (RPMI-HEPES complemented with 100uM xanthurenic acid, 200 uM hypoxanthine and 10% BSA, pH7.4) and incubated at 21 °C for 24 hours. After 24 hours, ookinetes were magnet**-**purified using 1ul monoclonal anti-P28 antibody 13.1^32^ coupled to 10ul magnetic beads (Dynabeads M-280 Sheep Anti-Mouse IgG) and resuspened in 500ul TRIzol (Invitrogen) and stored at −80 °C. Female to ookinete conversion rates for the three biological replicates were as follows: 65% 67% and 75%, respectively.

Genomic DNA was used as a control for the library preparation protocol. For this, asexual blood stage parasites were harvested via heart puncture, resuspended in 15 ml RPMI-HEPES, and passed through a Plasmodipur filter (Europroxima) to remove white blood cells. Parasite/RBC pellet was lysed with 10 ml RBC-lysis buffer (150 mM NH_4_Cl, 10 mM KHCO_3_, 1 mM EDTA) for 20 min on ice. Parasite-pellets were washed once in ice-cold PBS and lysed in buffer A (500 mM NaAc, 100 mM NaCl, 1 mM EDTA, pH5.2) and 3% SDS. DNA was extracted using phenol:chloroform and sheared to 150 bp fragments on a Bioruptor Plus sonication device (Diagenode).

### RNA sequencing

RNA was extracted using the Direct-zol RNA MiniPrep kit (Zymo). Residual gDNA was digested with the TurboDNA-free kit (Ambion). Stranded RNA sequencing libraries were prepared using the RNA HyperPrep Kit (KAPA) following manufacturer’s protocol with the exception of the amplification step set to 60 °C. The RNA library was sequenced in 43 bp paired-end reads on an Illumina NextSeq. 500 sequencer and each sample was split over four non-independent lanes. RNAseq was performed using biological triplicates for each condition.

We used sheared genomic DNA (gDNA) as a technical control and did not observe a bias towards GC-rich(er) sequences (Fig. S1A), a known problem in *Plasmodium falciparum* NGS^13^.

### RNA sequencing analysis

Quality of the reads were checked by eye using FASTQC. Reads were aligned separately for each lane to *PB*ANKA genome v3 using HiSat2 with default settings (v 2.0.5.2;–max intron size 5000;–fr)^83^. Mapped reads were pooled into one bam file per condition and replicate. Importantly, to adjust for potential GC-bias gDNA was processed alongside RNA samples as a control for the RNA library preparation. GC-bias was calculated according to^84^ using the default settings for *computeGCBias* in DeepTools2 suite^75^.

The PCA plot was calculated using default settings in the DeepTools2 suite^75^.

We calculated the FPKM (Fragments Per Kilobase of transcript per Million mapped reads) value for each gene using featurecounts with default settings^85^.

For comparative transcriptomics we used Deseq. 2 (v2.11.39) with default settings^86^. We considered significantly differently regulated genes as having a p value of lower than 0.001 (adjusted for multiple testing with the Benjamini-Hochberg procedure which controls false discovery rate (FDR)).

All of the analysis has been performed using usegalaxy.org (an open source, web-based platform for data intensive biomedical research)^87^ unless stated otherwise. Additionally, usegalaxy.eu was used for some calculations.

## Supplementary information

Supplementary information.

Supplementary figure S1.

Supplementary figure S2.

Supplementary figure S3.

Supplementary figure S4.

Supplementary figure S5.

Supplementary figure S6.

Supplementary table S1.

Supplementary table S2.

Supplementary table S3.

Supplementary table S4.

## Data Availability

The data discussed in this publication have been deposited in NCBI’s Gene Expression Omnibus^88^ and are accessible through GEO Series accession number GSE130278 (https://www.ncbi.nlm.nih.gov/geo/query/acc.cgi?acc=GSE130278). This includes raw read data files as well as output from Deseq. 2^86^ and featurecounts^85^. Bam files are deposited on zenodo.com.
