# Supplementary material for: An epigenetic map of malaria parasite development from host to vector
Source: Sci Rep. 2020 Apr 14;10:6354. doi: 10.1038/s41598-020-63121-5 (PMC7156373; doi:10.1038/s41598-020-63121-5)
Supplement: Supplementary file 1 — Supplementary information. [file 41598_2020_63121_MOESM1_ESM.pdf]

An epigenetic map of malaria parasite development from host to vector

Supplementary information

Kathrin Witmer[1]*, Sabine AK Fraschka[2,3], Dina Vlachou[1], Richárd Bártfai[2], George K Christophides[1]*

[1]Department of Life Sciences, Imperial College London, SW7 2AZ, London, UK

[2]Department of Molecular Biology, Radboud University, 6525 GA, Nijmegen, The Netherlands

[3] Current address: Institute of Medical Genetics and Applied Genomics, University of Tübingen, 72076 Tübingen, Germany

* corresponding authors

Email: k.witmer@imperial.ac.uk, kathrin.witmer@gmail.com (KW)

Email: g.christophides@imperial.ac.uk (GKC)

S1 Fig. RNA sequencing of *P. berghei* ABS, FG, MG and OOK.

(A) No GC-bias according to [1] was detected when processing genomic DNA alongside the RNA samples. Data was analysed in non-overlapping 300bp bins and the number of reads according to the underlying GC-percentage of the regions is plotted (upper boxplot). Lower panel shows read counts normalised to the expected read counts according to the underlying GC-value.

(B) Principal component analysis of aligned RNAseq data of all *P. berghei* life stages investigated including the gDNA control. Aligned and sorted reads of each sample were grouped into non-overlapping 1000bp bins. Triplicates cluster together, and transcriptionally, male gametocytes are similar to asexual blood stage parasites, whereas female gametocytes are more closely related to ookinetes.

S2 Fig. A unique heterochromatic dynein gene in *P. berghei*.

(A) Heat map of all 22 dynein genes and their respective H3K9ac occupancy and relative transcripts for each life cycle stage. H3K9ac enrichment for each gene locus is shown as log_2-transformed H3K9ac ChIP/input (1000bp upstream of the ATG, the ORF, and 500bp downstream of the stop codon, respectively). Pink colour indicates relative transcripts (in FPKM).

(B) Bar plot of the left arm of PbANKA chromosome 6. Boxes represent genes and arrows indicate the orientation for each gene. Open boxes indicate neighbouring genes. Peaks correspond to log_2 transformed data of either *Pb*HP1-ChIP/input or H3K9ac-ChIP/input. Log_2 scale for *Pb*HP1 is -3 to 3 and -2 to 2 for H3K9ac, respectively. Numbers indicate relative transcripts (in FPKM) for each stage.

S3 Fig. Variant expression of two genes according to the Malaria Cell Atlas [2]

(A) PCAs of *PbANKA_0300600* for asexual blood stages, male and female gametocytes, and ookinetes (shown together with oocysts). Each symbol represents a cell, and different shapes indicate different developmental stages. The cells are coloured by *PbANKA_0300600* expression. *PbANKA_0300600* shows mostly stage-specific expression during the intraerythrocytic developmental cycle, while it shows variegated expression in both male and female gametocytes and to a lesser degree in ookinetes. This is representative of our observation from Fig. 2, where this genes is marked by both euchromatin and heterochromatin in all stages investigated.

(B) PCAs of *PbANKA_1300500* (ε tubulin) for asexual blood stages, male and female gametocytes and ookinetes (shown together with oocysts). Each symbol represents a cell, and different shapes indicate different developmental stages. The cells are coloured by *PbANKA_1300500* expression. *PbANKA_1300500* is mainly expressed in male gametocytes and to some extent female gametocytes.

S4 Fig. The TSS of ribosomal protein genes is not closer to the ATG than for other genes in *P. falciparum*.

Boxplots showing the distance of the middle of the TSS mapped by Adjalley et al.[3] to the ATG for each ribosomal protein gene and for all other genes in *P. falciparum*. There is no statistical difference between the two gene groups (unpaired t-test, $P>0.1$). Whiskers indicate the 10 and 90 percentiles, respectively. The median is indicated within the box. The median distance between the middle of the TSS to the ATG for ribosomal protein genes and other genes is 117bp and 166bp, respectively.

S5 Fig. H3K9ac occupancy in female gametocytes in different gene sets.

(A) Heatmap showing H3K9ac occupancy of the 5'UTR of translationally repressed genes (DOZI-controlled) [4] compared to all euchromatic genes.

(B) Box plot showing mean 5'UTR values from the two gene groups from (A). DOZI-controlled genes have significantly less H3K9ac coverage in their 5'UTR. Asterisks mark significance (Mann-Whitney test, p =0.0004).

(C) Heatmap showing H3K9ac occupancy of the 5'UTR of AP2-O-controlled genes [5] compared to all euchromatic genes.

(D) Box plot showing mean 5'UTR values from the two gene groups from (C). No statistically significant difference has been found (Mann-Whitney test (ns), p=0.6245).

S6 Fig. Heterochromatin distribution in asexual blood stages is very similar to the *Pb*ANKA isolate used in Fraschka et al. (2018).

(A) Chrosomosomal location of each heterochromatic gene from this study and the study from Fraschka and colleagues. Each filled dot represents a gene. Colour code shows if a gene was found to be heterochromatic in both studies (blue), this study (green) or the study from Fraschka et al [6] (red). Taken both studies together, only four heterochromatic genes are not located at subtelomeric regions (highlighted with an arrow).

(B) and (C) Bar chart of two of the genes highlighted in A. Small arrows underneath the gene indicates direction of transcription. Log_2 scale for *Pb*HP1/input ratio is (-3 to 3).

S1 Table. Summary of RNA-seq and ChIP-seq alignment statistics

S2 Table. *Pb*HP1 enrichment and relative transcripts of heterochromatin genes.

Mean *Pb*HP1 values for all genes found to be enriched by *Pb*HP1. FPKM (mean of three biological replicates) is shown.

S3 Table. List of genes that are significantly up- and downregulated in FG compared to all other stages.

Genes used for Figure 5.

S4 Table. GO-terms associated with up- or downregulated genes in female gametocytes.

GO and GO slim terms of genes used in Table S3, as shown in Figure 5B.

References

1. Benjamini, Y. & Speed, T. P. Summarizing and correcting the GC content bias in high-throughput sequencing. *Nucleic Acids Res.* **40**, e72–e72 (2012).
2. Howick, V. M. *et al.* The Malaria Cell Atlas: Single parasite transcriptomes across the complete Plasmodium life cycle. *Science (80-.).* **365**, eaaw2619 (2019).
3. Adjalley, S. H., Chabbert, C. D., Klaus, B., Pelechano, V. & Steinmetz, L. M. Landscape and Dynamics of Transcription Initiation in the Malaria Parasite Plasmodium falciparum. *Cell Rep.* **14**, 2463–2475 (2016).
4. Guerreiro, A. *et al.* Genome-wide RIP-Chip analysis of translational repressor-bound mRNAs in the Plasmodium gametocyte. *Genome Biol.* **15**, 493 (2014).
5. Kaneko, I., Iwanaga, S., Kato, T., Kobayashi, I. & Yuda, M. Genome-Wide Identification of the Target Genes of AP2-O, a Plasmodium AP2-Family Transcription Factor. *PLoS*

Pathog. **11**, e1004905 (2015).

6. Fraschka, S. A. *et al.* Comparative Heterochromatin Profiling Reveals Conserved and Unique Epigenome Signatures Linked to Adaptation and Development of Malaria Parasites. *Cell Host Microbe* **23**, 407-420.e8 (2018).
