# Supplementary figures and images for: An epigenetic map of malaria parasite development from host to vector
Source: Sci Rep. 2020 Apr 14;10:6354. doi: 10.1038/s41598-020-63121-5 (PMC7156373; doi:10.1038/s41598-020-63121-5)

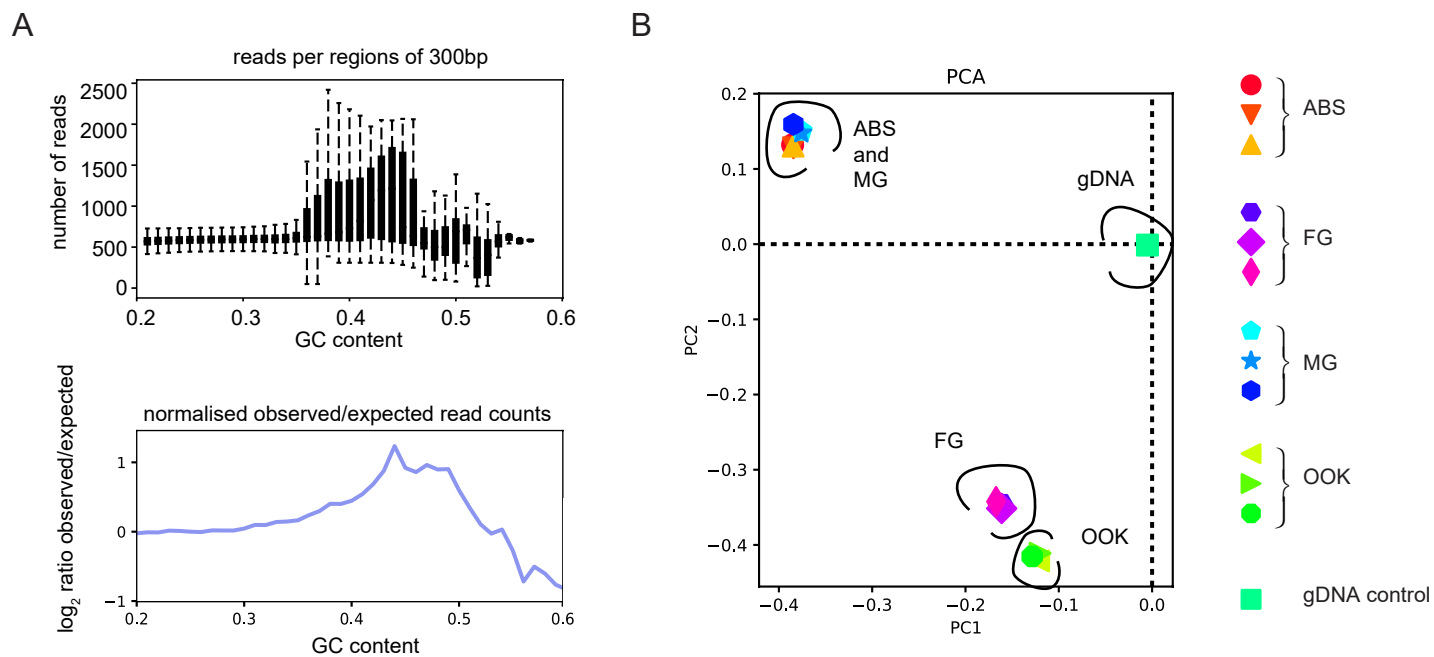

Figure S1

Supplement: Supplementary file 2 — Supplementary figure S1. [file 41598_2020_63121_MOESM2_ESM.pdf]

**A**

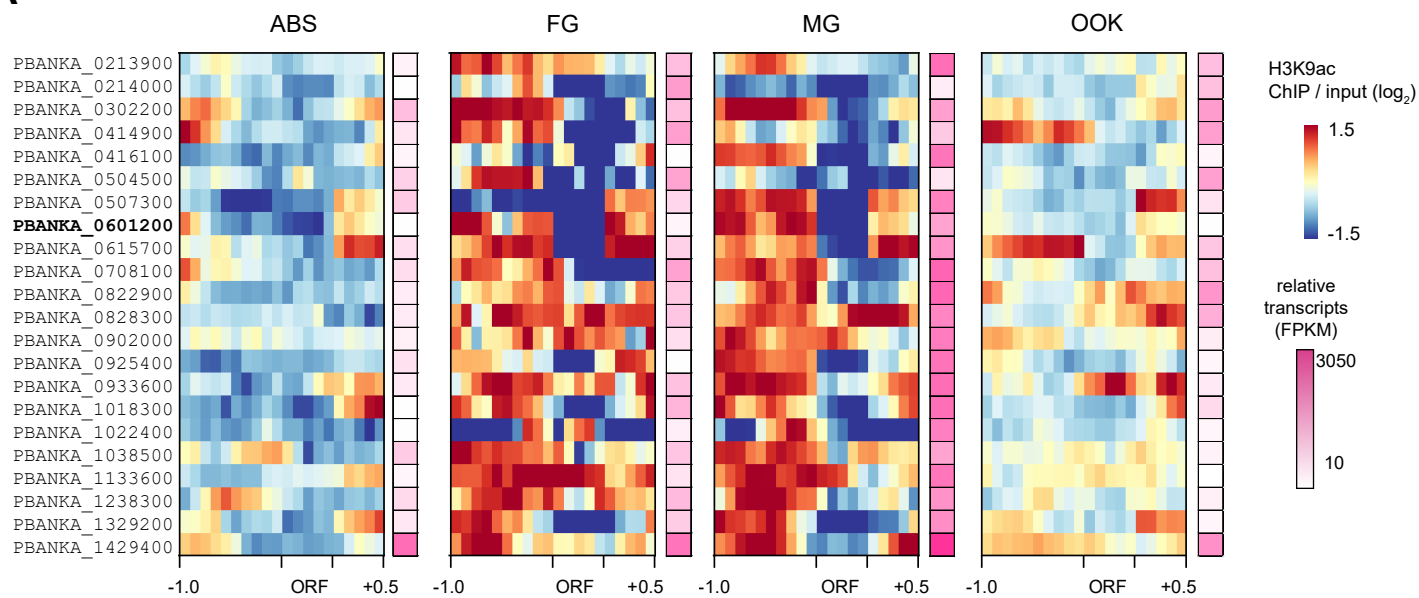

**B**

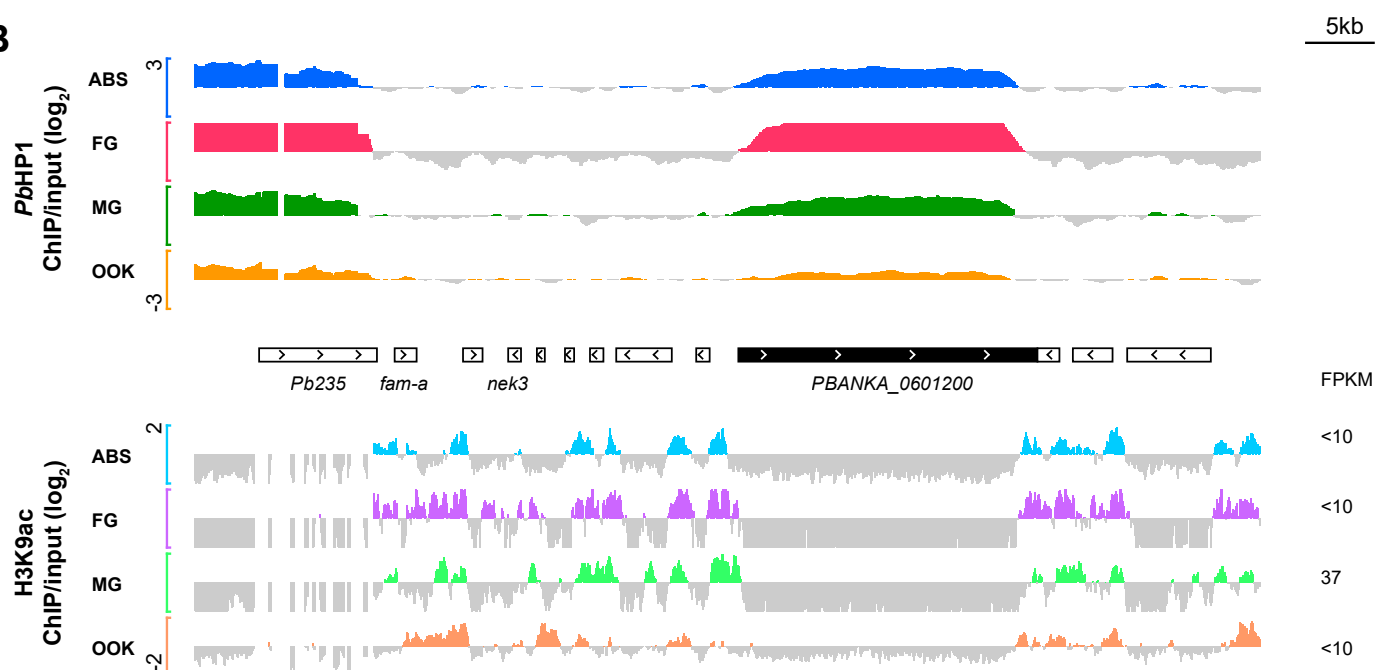

Figure S2

Supplement: Supplementary file 3 — Supplementary figure S2. [file 41598_2020_63121_MOESM3_ESM.pdf]

A

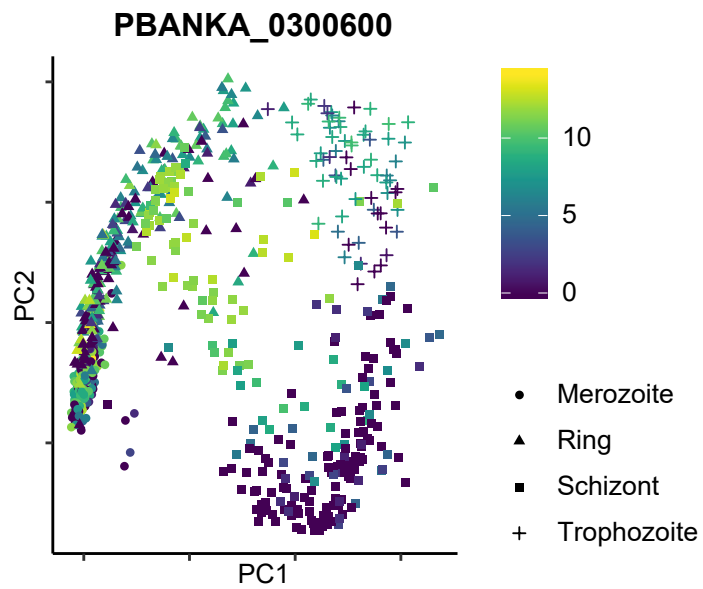

B

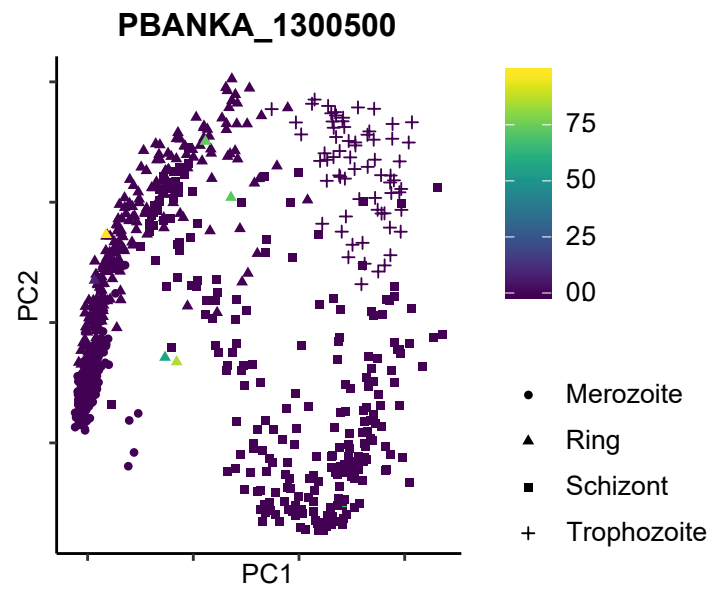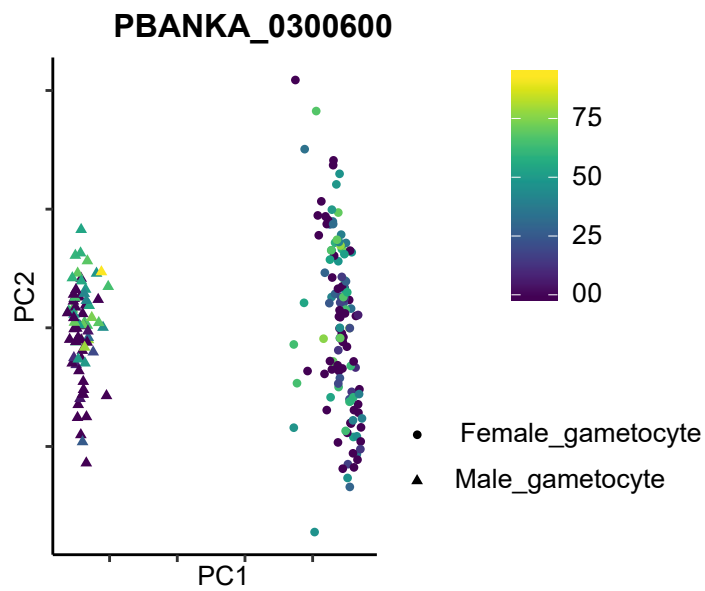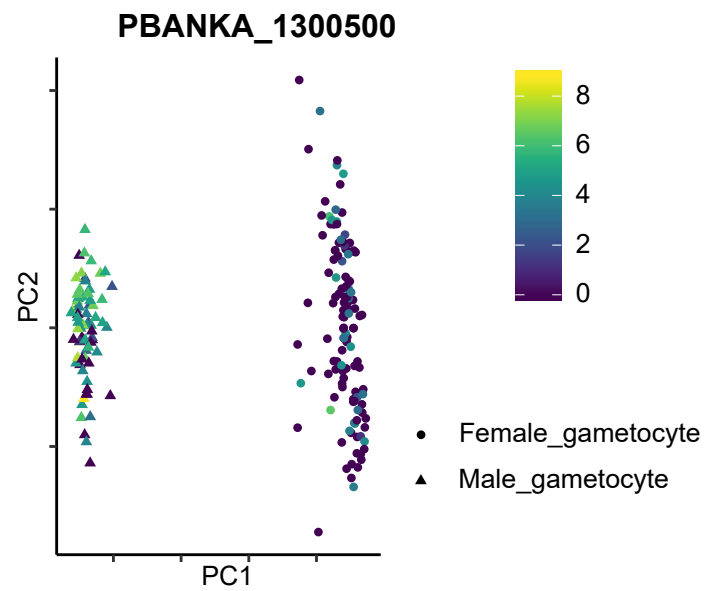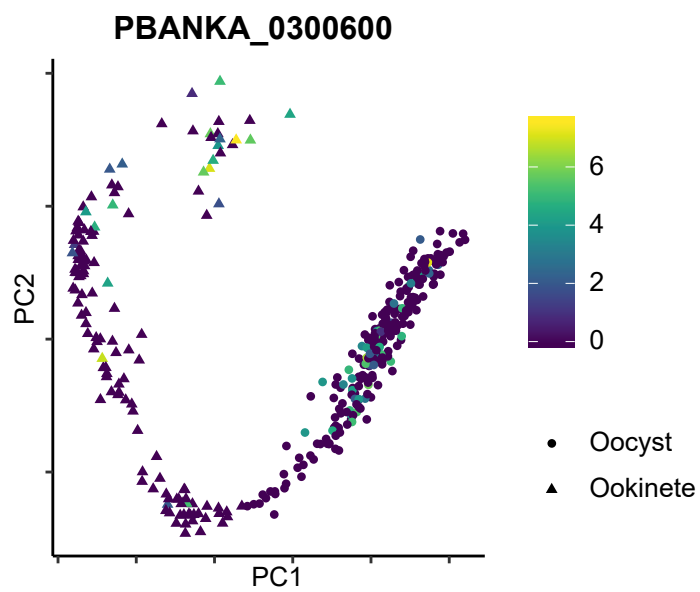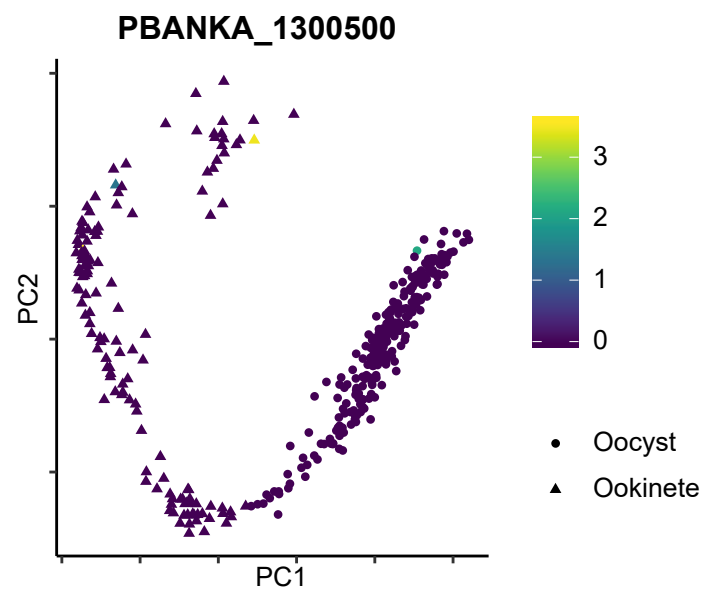

Figure S3

Supplement: Supplementary file 4 — Supplementary figure S3. [file 41598_2020_63121_MOESM4_ESM.pdf]

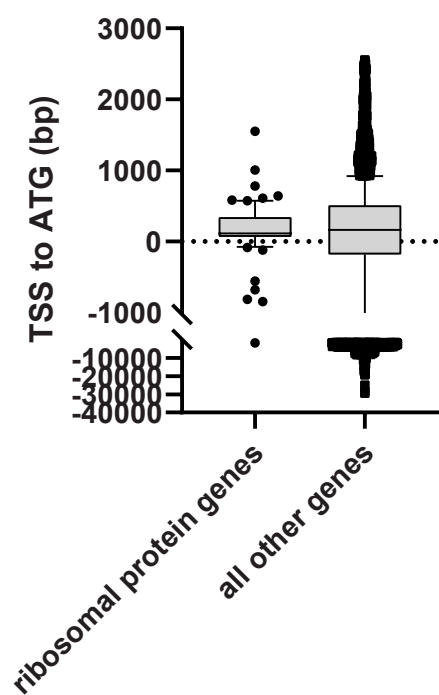

Figure S4

Supplement: Supplementary file 5 — Supplementary figure S4. [file 41598_2020_63121_MOESM5_ESM.pdf]

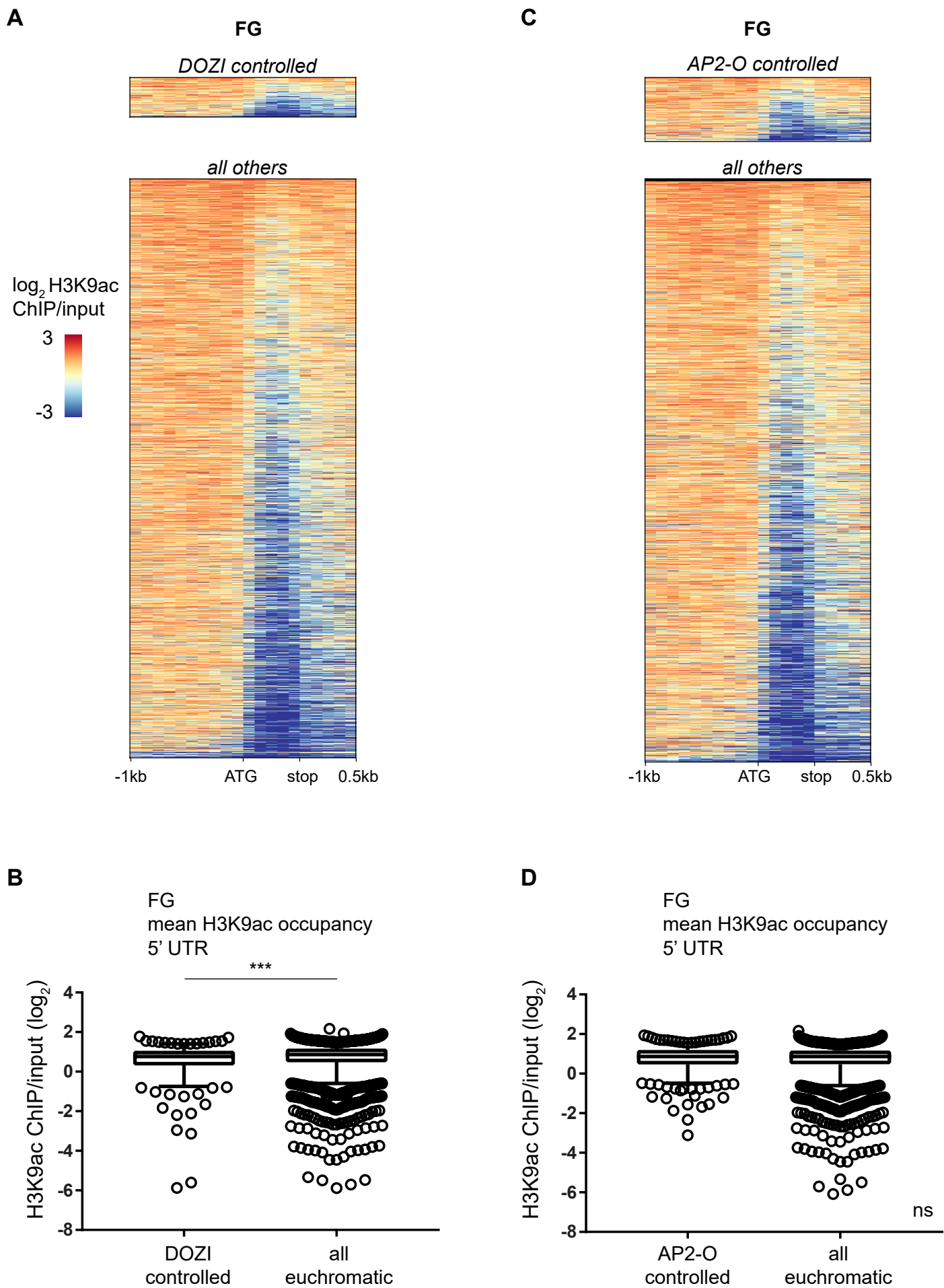

Figure S5

Supplement: Supplementary file 6 — Supplementary figure S5. [file 41598_2020_63121_MOESM6_ESM.pdf]

A

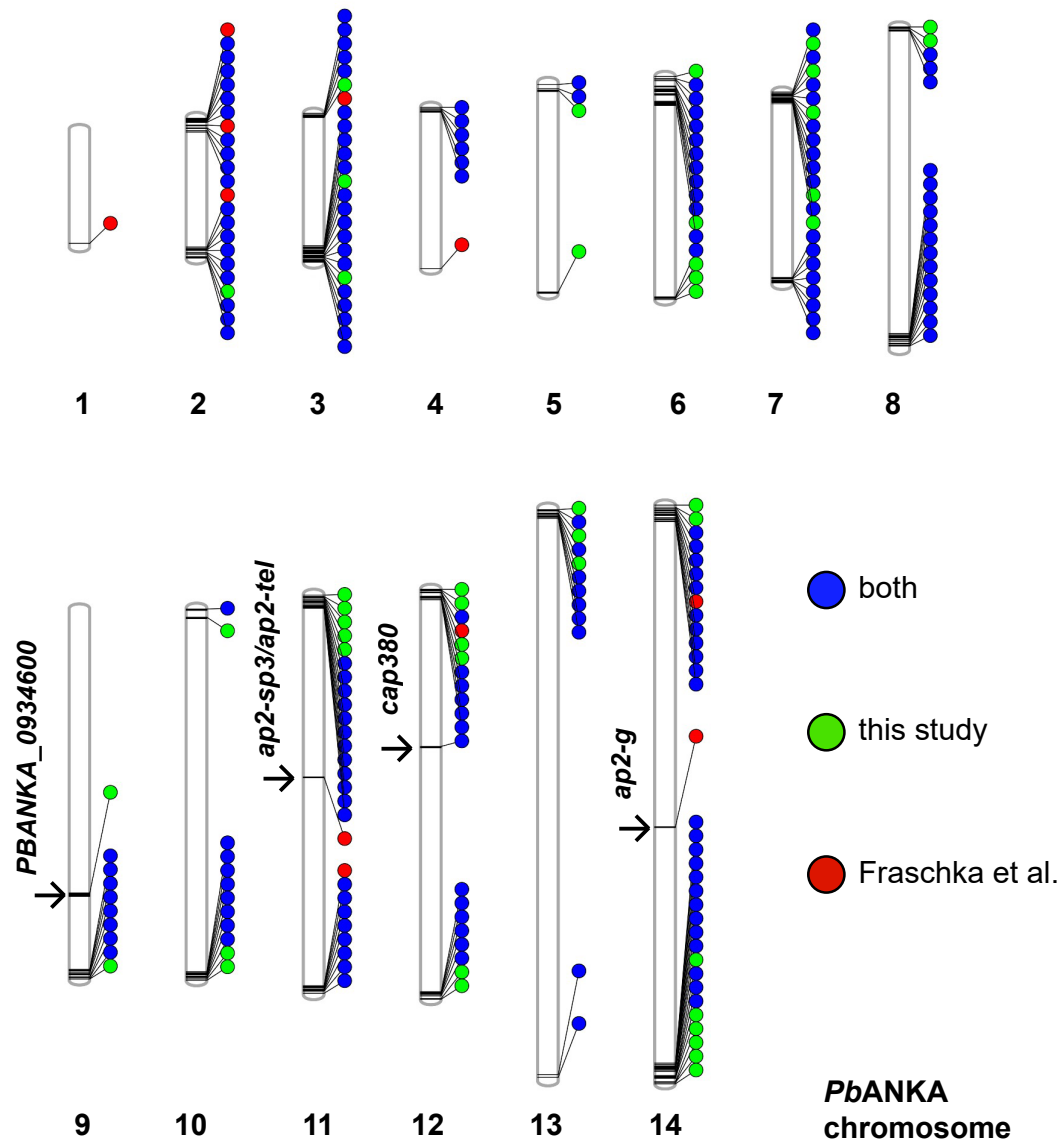

B

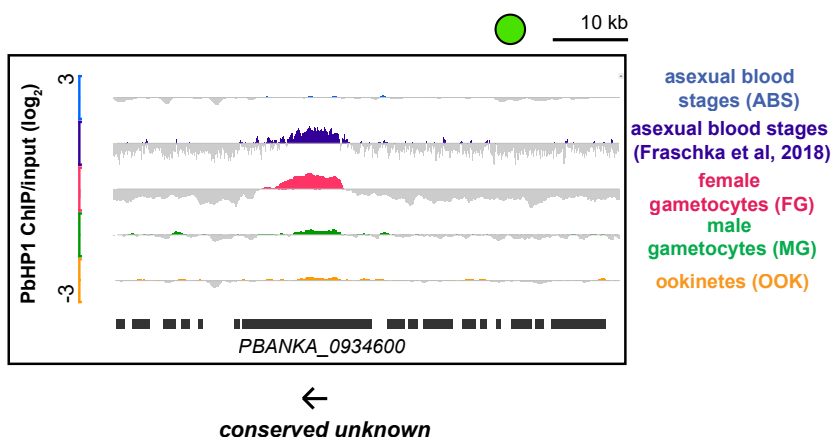

C

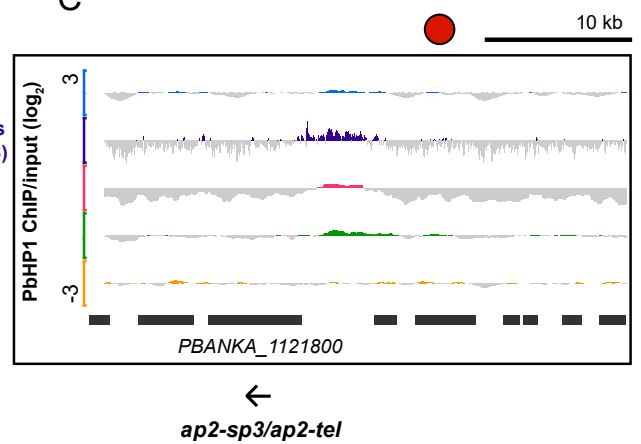

Figure S6

Supplement: Supplementary file 7 — Supplementary figure S6. [file 41598_2020_63121_MOESM7_ESM.pdf]
